# Novel Deep Eutectic Solvent-Based Protein Extraction Method for Pottery Residues and Archeological Implications

**DOI:** 10.1021/acs.jproteome.2c00340

**Published:** 2022-10-21

**Authors:** Manasij Pal Chowdhury, Cheryl Makarewicz, Henny Piezonka, Michael Buckley

**Affiliations:** † Manchester Institute of Biotechnology, University of Manchester, 131 Princess Street, Manchester M1 7DN, U.K.; ‡ Interdisciplinary Centre for Ancient Life, Department of Earth and Environmental Sciences, University of Manchester, Oxford Road, Manchester M13 9PL, U.K.; § Institute for Prehistoric and Protohistoric Archaeology, Kiel University, Johanna-Mestorf Strasse 2-6, Kiel D-24118, Germany; ∥ Cluster of Excellence ROOTS: Social, Environmental, and Cultural Connectivity in Past Societies, Kiel University, Leibniz Strasse 1, Kiel 24118, Germany

**Keywords:** Deep eutectic solvent, DES, Archaeological pottery, Residue analysis, Protein extraction, Cross-species proteomics

## Abstract

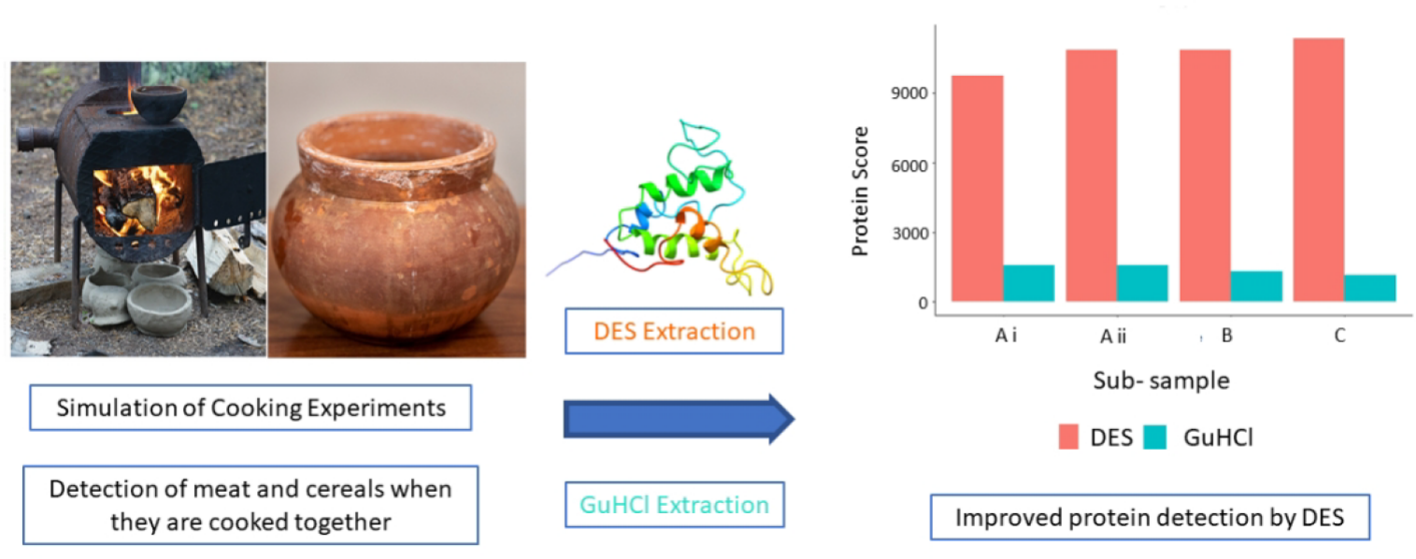

Proteomic analysis of absorbed residues is increasingly used to identify the foodstuffs processed in ancient ceramic vessels, but detailed methodological investigations in this field remain rare. Here, we present three interlinked methodological developments with important consequences in paleoproteomics: the comparative absorption and identification of various food proteins, the application of a deep eutectic solvent (DES) for extracting ceramic-bound proteins, and the role of database choice in taxonomic identification. Our experiments with modern and ethnoarcheological ceramics show that DES is generally more effective at extracting ceramic-bound proteins than guanidine hydrochloride (GuHCl), and cereal proteins are absorbed and subsequently extracted and identifiedat least as readily as meat proteins. We also highlight some of the challenges in cross-species proteomics, whereby species that are less well-represented in databases can be attributed an incorrect species-level taxonomic assignment due to interspecies similarities in protein sequence. This is particularly problematic in potentially mixed samples such as cooking-generated organic residues deposited in pottery. Our work demonstrates possible proteomic separation of fishes and birds, the latter of which have so far eluded detection through lipidomic analyses of organic residue deposits in pottery, which has important implications for tracking the exploitation of avian species in various ancient communities around the globe.

## Introduction

Over the last few decades, organic residue analysis (ORA), including compound-specific isotope analysis of organic residues deposited in ancient ceramic vessels, has revealed crucial information on different types of food processed by people previously undetectable through the zooarcheological and paleobotanical records.^1,2^ Although lipidomic analysis has traditionally been the tool of choice for ORA, proteomic techniques are now coming forth as a viable approach that offers opportunities to obtain species-specific information on various foodstuffs processed by people for their consumption. Proteomic analysis has been used to identify the consumption of milk and milk products^3,4^ and fish roe,^5^ as well as cereals, legumes and pulses.^6−9^ The identification of cereal processing and consumption through proteomics is potentially of pivotal importance, as lipidomic analysis has largely been limited to the identification of animal products^10^ and lipid-based biomarkers have been identified for relatively few cereals and other plant-based food.^11−13^

Experimental investigations documenting the behavior of various organic compounds during cooking processes and their subsequent degradation allow us to identify how particular compounds can act as species-specific biomarkers. However, in spite of the increasing importance of proteomic studies on archeological ceramics and its recent use for species-specific identification of resources,^5−7^ information on the comparative absorption and subsequent survival of different proteins and the level of accuracy with which proteomics can be used for species-specific identification of various resources remains surprisingly sparse. Furthermore, extraction methods and reagents for protein recovery from archeological ceramic residues remain somewhat limited at this early stage of development, potentially undercutting the analytical power of proteomic analyses as a means to understand ancient food processing habits.

The protein extraction process is a key bottleneck in archeological research, owing to the strong forces of attraction between proteins and the ceramic matrix, which, although aiding their preservation, makes their extraction difficult. Consequently, a variety of reagents have been tried for dislodging the proteins from ceramics, including trifluoroacetic acid,^14^ hydrofluoric acid,^15,16^ sodium dodecyl sulfate (SDS) solution in conjunction with commercial M-PER (Mammalian Protein Extraction Reagent, Thermo Fisher; used in a gel-assisted sample preparation technique^8,17^), and guanidine hydrochloride (GuHCl).^18^ However, very few experiments have systematically compared the efficiency of various available extraction reagents and aimed at developing an optimum methodology for the extraction of proteins from ceramics (apart from works by Barker and co-workers involving protein-spiked ceramics^19,20^).

Deep eutectic solvents (DESs) are a mixture of Lewis or Bronsted acids and bases, which form a eutectic mixture with a melting point much lower than the melting points of the constituent compounds^21,22^ (Supplementary Figure S1). They were introduced as an alternative to ionic liquids and form a relatively new area of research, with the first report of a DES liquid published only this century.^23^ Most common DESs are a mixture of a quaternary ammonium salt with either a metal salt or a hydrogen bond donor.^21,22^ The large scale charge delocalization caused by hydrogen bonding between the hydrogen bond donor and the anions acting as a hydrogen bond acceptor reduces the electrostatic force of attraction between the cations and anions, resulting in a large freezing point depression.^22,24^ DESs have been used as reaction media for both inorganic and organic synthesis, biomass processing and biodiesel production, electroplating and electrodeposition of metals, assembly of metal-organic-frameworks (MOFs), and production of various nanoparticles and as chromatographic media and extraction reagents. Recently, DESs have also been used for protein extraction,^25,26^ with reports of successful extraction from oilseed cakes^27^ and pomegranate peel,^28^ extraction of keratin from rabbit hair^29^ and wool,^30^ and extraction of collagen from cod skin.^31^ Although there are various comprehensive reviews of the applications of DESs available in literature,^32−34^ to date, no studies have looked at comparing the efficiency of DES and conventional GuHCl solution for the extraction of proteins from archeological ceramics.

In order to resolve the above-mentioned methodological questions, we conducted cooking experiments involving known plant- and animal-based foodstuffs under experimental and ethnographic conditions using modern ceramic vessels to address three major aspects of proteomic analysis of absorbed residues in ceramics, namely, development of better extraction protocols, the relative absorption of various food proteins, and the importance of database choice in the accuracy of species-specific identification of various resources using proteomics. We cooked cereals (wheat and barley), meat (minced beef and pork), and milk together in ceramic vessels to determine the consilience between the known foods that went into the pots and the proteomics-based taxonomic identification of residues absorbed in the ceramic vessels. We also compared a DES-based protein extraction method to the standard GuHCl-based extraction approach and investigated how varying time, temperature, and the use of ultrasonication can influence the retrieval of proteins. This was applied to ceramics that were artificially spiked with protein and also to two sets of ceramics in which different cooking experiments were simulated. In order to investigate how database choice and the evolutionary diversity of the proteins under consideration affect protein identification and their taxonomic assignment, we also analyzed proteins recovered from residues generated by fish and bird tissues cooked under ethnoarcheological conditions. High-resolution taxonomic identification of residues would help better establish the resources exploited by ancient humans for their subsistence, with particularly important implications for tracing food use patterns in regions of the world where animals typically underrepresented in the zooarcheological record, including birds and fish, may have been important constituents of their food cultures.

The Late Glacial to Early Holocene Stone Age in North Eurasia is defined by some of the world’s earliest examples of ceramic vessels produced and used by hunter–gatherers.^35,36^ In Siberia and adjacent regions (e.g., Japan, the Baltic region), this period has often been termed an “aquatic” Neolithic, defined by a primary focus on the exploitation of freshwater fishes abundant in the lakes, streams, and rivers throughout the region.^37,38^ However, recent lipid residue analyses of materials from various parts of North Eurasia suggest diverse regional patterns in resource use, involving, for example, the exploitation of nonruminant animals along with aquatic resources in the Southeastern Baltic region.^39−41^ Other animal resources may have also been exploited as sources of fat and meat but are to date not readily detectable in lipid residues. Fish, water fowl, and forest fowl are also readily available in the taiga and forest steppes of western Siberia and, even today, play an important role in the daily subsistence of indigenous groups inhabiting this region. However, while fish appear to have been regularly exploited by ancient hunter–gatherer groups in these environments, identifying water fowl and forest fowl, highly reliable seasonal resources that can be stored by drying or freezing,^42^ has proved so far elusive.

## Experimental Section

### Materials and Extraction Methods

Three sets of experimental pottery were prepared for this study. The first set included ground ceramics spiked with bovine serum albumin (BSA) solution to determine the feasibility of DES as compared to GuHCl as an extracting agent as well as for studying the effects of temperature, ultrasonication, and time on the extraction procedure. Once the optimal conditions for both GuHCl- and DES-based extraction were established, we used two additional sets of experimental ceramics, one in which a mixture of meat (beef and pork), milk, and cereals was cooked and another in which bird or fish tissue was cooked to understand the absorption and subsequent identification of various proteins and to better compare the efficiency of GuHCl and DES as extraction agents.

### Preparation of BSA-Spiked Ceramics

Commercially obtained unglazed pots (750 mL, 13.5 cm diameter × 9 cm high; Terracotta World, Otley, U.K.) were used to prepare artificially aged BSA-spiked ceramic–protein mixtures using a modified approach based on the method of Craig and Collins.^18^ The ceramics were ground to a fine powder using a pestle and mortar, and 40 g of ground ceramics was added to 200 mL of 1% BSA solution. The mixture was heated at 85 °C for 7 d with continuous stirring, with distilled water being added to compensate for evaporated water every day. After 7 days, the resultant slurry was centrifuged, and the supernatant liquid was removed. The residue was washed with 50 mL of distilled water and dried for subsequent protein extraction.

### Meat, Cereals, and Milk Cooking Experiments in Modern Ceramics

A set of ceramic pots (Pots A–E) were obtained as above and washed with distilled water. Subsequently, 500 mL of tap water (Manchester City Council, supplied by United Utilities and obtained from Lake District and local reservoir sources; soft water; 2.24 Hardness Clarke) was added and heated to boiling, following which the water was discarded. Once the pot was dried, a further 250 mL of tap water was added and heated to boiling. To it, a mixture of 50 g each of beef mince (20% fat, 18.3 g protein per 100 g, Lidl, UK), pork mince (20% fat, 22.5 g protein per 100 g, Sainsburys UK), crushed pearl barley (2.7 g protein per 100 g, Sainsburys, UK), and crushed wheat (11.9 g protein per 100 g, Sofra Jarish cracked wheat) was added, along with 50 mL of whole milk (3.5 g protein per 100 mL, Sainsburys, UK). The mixture was simmered for 60 min, with periodic stirring and addition of water to ensure that it did not burn. After simmering, the contents of the pot were discarded, the pot was rinsed with tapwater, and any food or charred residue sticking to it was scraped and discarded. For pots A, D, and E, the cooking process was repeated 20 times, and for pots B and C, it was repeated 25 times.

### Fish and Bird Cooking Experiments in Prehistoric Pot Reproductions

**Figure 1:**
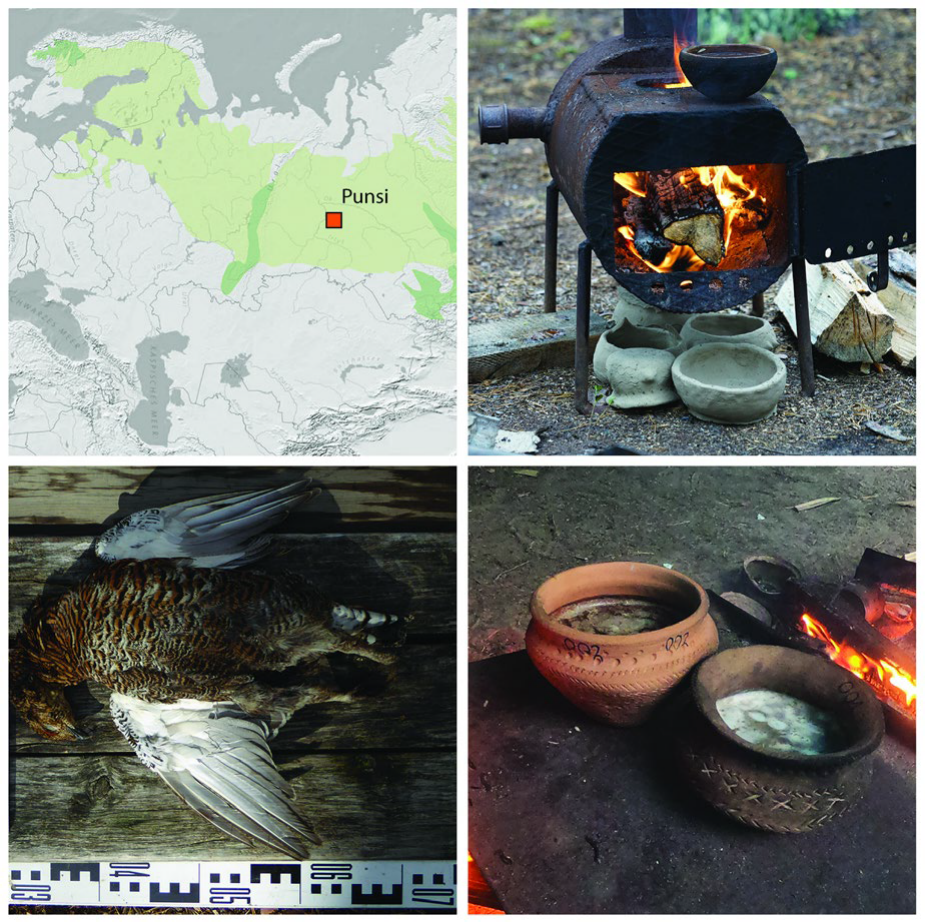
Map showing the location of Punsi and the details of the experiments. From top left (clockwise), the location of Punsi in Western Siberia, Russia; the traditional wood-fired ovens used for the cooking experiments; ceramic pots showing the result of the cooking experiments; and a black grouse that was hunted and used for the cooking experiment.

A set of cooking experiments involving the processing of fish and bird tissues in six ceramic pots (Pots 1–6, reproductions of prehistoric pottery) were conducted at Yurty Punsi (a summer settlement of the Yugan Khanty indigenous community who partly maintain a seasonal mobile lifestyle based on hunting and fishing^43^), Western Siberia, in the summer of 2019 (Figure 1). Yugan Khanty traditional subsistence includes hunting of wild game birds, fishing with stationary devices, the collection of wild plant foods and, until recent years, was also supplemented by small-scale reindeer herding for transport.^44,45^

The Khanty community, who caught fish and fowl for their own meals, provided the samples used for the cooking experiments (Table 1). Pike was caught from Lake Bolshoe Kayukovo using fishing rods; the other fish were taken from fishtraps placed in the same lake, and the ducks and black grouse were hunted by shooting. The pottery vessels used for the experiment included prefabricated replicas of archeological pottery and an additional vessel made for this experiment; the pottery had never been previously used (Table 1). Fish and fowl samples were first disarticulated in metal and plastic bowls provided by the Khanty, and cleaned with soap and water drawn from a well. The cooking experiments took place in the open air at Punsi settlement on wood-fired traditional metal ovens previously used in mobile dwellings during nomadic episodes (Figure 1) in which 30–40 g of materials were placed in the respective pots (as detailed in Table 1) and simmered in ∼250 mL of clean water for an hour, stirring every 10–15 min.

**Table 1:** Six Pottery Vessels Used for Cooking Fish and Meat and the Materials Cooked in Them during the Experiment

ceramic pot no.	vessel type used	material cooked
POT1	prefabricated replica of prehistoric vessel	one fin and skin of pike (*Esox lucius*), almost no fat (repeated 10 times)
POT2	prefabricated replica of prehistoric vessel	black grouse (*Lyrurus tetrix*), meat of upper and lower breast (repeated 10 times)
POT3	prefabricated replica of prehistoric vessel	crucian carp (*Carassius carassius*) (repeated 10 times)
POT4	vessel handmade for the experiment	ide (*Leuciscus idus*) (repeated 9 times)
POT5	prefabricated replica of prehistoric vessel	northern pintail duck (*Anas acuta*) (repeated 10 times)
POT6	prefabricated replica of prehistoric vessel	duck (Russian Chirok) (repeated 7 times)

### Protein Extraction

#### Protein Extraction from BSA-Spiked Ceramics

The BSA-spiked ceramics were used for a pilot study to explore the feasibility of the DES as an alternative for the traditional GuHCl-based extraction protocol. Eight different combinations of extraction agents and conditions were analyzed. GuHCl solution (6 M) was used to extract the proteins at 65 °C for 4 h with and without ultrasonication, and at 4 °C for 66 h without ultrasonication, whereas the urea–GuHCl DES was used for extraction at 65 °C for 2, 4, 6, and 8 h with ultrasonication as well as for 4 h without ultrasonication. For each condition, 1.5 g aliquots of the prepared BSA–ceramic mixture were analyzed in triplicate, along with one blank (Supplementary Table S1).

#### Protein Extraction from BSA-Spiked Ceramics Using 6 M GuHCl

For extraction of the BSA-spiked ceramics using 6 M guanine hydrochloride solution, 5 mL of the reagent was added to an accurately weighed amount of ground ceramics. After vortexing, the mixture was kept at a specific temperature depending on the condition. After the relevant time elapsed, the mixture was centrifuged, and the clear supernatant liquid was decanted and ultrafiltered using 3 kDa molecular weight cutoff (Pall Corporation, New York) filters. To the residue, 2 mL of 50 mM ammonium bicarbonate (ABC) was added and ultrafiltered, and the process was repeated twice. The residue after ultrafiltration was dissolved in 50 mM ammonium bicarbonate to make the total volume 1 mL and the amount of protein was measured using a Qubit 2.0 fluorometer (see Supplementary Table S1 for the masses of the ceramics analyzed and the amount of BSA recovered).

#### Protein Extraction from BSA-Spiked Ceramics Using DES

The DES solvent chosen for the experiment was urea and guanidine hydrochloride (GuHCl) in a 2:1 molar ratio.^24^ The compounds were weighed, mixed, and heated at ∼70–75 °C until a homogeneous clear liquid was obtained. The mixture was then stored at room temperature until the time of use, at which point it was liquefied by heating at 70 °C. Five milliliters of the DES in the liquid state was measured and added to the ground ceramics, and after the relevant time elapsed, the mixture was immediately poured (while still in liquid phase) to a centrifuge tube, and 5 mL of distilled water was added to it. The remaining residue was extracted using 5 mL of distilled water and the combined mixture was centrifuged. The supernatant liquid was ultrafiltered and redissolved into ABC, and the amount of BSA was measured as described above.

#### Extraction of Proteins from Experimental Ceramics Using 6 M GuHCl

For the experimental ceramics, parts of the pot were crushed, and the ground ceramic mixture was divided into triplicates for extraction using the two methods. Proteins were extracted from ∼4–4.5 g of ceramic powder using 6 M GuHCl assisted by ultrafiltration. GuHCl (5 mL of 6 M) was added to the ground ceramics (refer to Supplementary Tables S2 and S3 for the mass of each sample analyzed), and the mixture was vortexed to ensure mixing. The mixture was incubated at 4 °C for 66 h and then centrifuged at 7500 rpm for 15 min. The supernatant liquid was decanted and stored, and 2 mL of deionized water was added to the solid residue. The mixture was vortexed and centrifuged for 7500 rpm for 15 min, following which the supernatant liquid was decanted and the residue discarded. The combined supernatant liquid was ultrafiltered using Pall 3 kDa ultrafilters. To the retentate, 3 mL of 50 mM ammonium bicarbonate (ABC) was added and ultrafiltered, the ultrafiltration was repeated twice, and the retentate was redissolved into 500 μL of 50 mM ABC. The mixture was then reduced by addition of 21 μL of 100 mM dithiothreitol (DTT; 45 min at room temperature), and then alkylated with 42 μL of 100 mM iodoacetamide (IAM; 45 min at room temperature in the dark), and the alkylation was subsequently quenched by the addition of 21 μL of 100 mM DTT. The resultant protein solution was then digested using 0.4 μg of trypsin (Promega) at 37 °C for 18 h. The resultant tryptic peptides were desalted using OMIX C18 pipette tips with 50% acetonitrile (ACN) and 0.1% trifluoroactetic acid (TFA) as the eluent, and the eluate was dried and stored at −20 °C for proteomic analysis.

#### Extraction of Proteins from Experimental Ceramics Using DES

For DES extraction, approximately 4.5 g of ground ceramics were weighed accurately, and to it 5 mL of the DES solution was added (Supplementary Tables S2 and S3). The mixture was quickly vortexed, and the sample was incubated in an ultrasonic bath for 4 h at 65 °C with ultrasonication. The resultant liquid slurry was poured into 5 mL of distilled water, and an additional 5 mL of distilled water was added to the remaining residue and vortexed, and both of the resultant slurries were combined. The mixture in the falcon tube was then centrifuged at 7500 rpm for 15 min and the supernatant liquid was decanted and stored. To the remaining residue, 2 mL of distilled water was added, the mixture was vortexed to ensure mixing, and the resultant mixture was centrifuged. The supernatant liquid was then decanted and combined with the previously stored liquid. The solution was then ultrafiltered, redissolved into 50 mM ABC and trypsin digested (after reduction and alkylation), and the tryptic digest was desalted and prepared for LC-MS/MS analysis as described above.

#### LC-MS/MS Proteomic Analysis

Shotgun proteomics was used to identify the proteins extracted from the ceramics using LC-MS/MS. The dried tryptic peptides were reconstituted by dissolving in 5% ACN + 0.1% formic acid (FA) and analyzed using an UltiMate 3000 Rapid Separation LC coupled with an Orbitrap Elite mass spectrometer. The peptides were first concentrated on a precolumn (20 mm × 180 μm), followed by separation using a 1.7 μm Waters nanoAcquity Ethylene Bridged Hybrid C18 analytical column of 75 mm × 250 μm i.d. Gradient elution was used, beginning at 99% buffer A (0.1% FA in H_2_O)/1% buffer B (0.1% FA in ACN) and finishing at 75% buffer A/25% buffer B.

#### Data Analysis

Thermo ExtractMSN was used to convert the resultant RAW files into .mgf files, considering all precursor charges and the minimum and maximum precursor mass set at 600 and 3500 respectively. The grouping tolerance was set at 1.4, 1 intermediate scans were considered, with a minimum of 1 scan/group, a minimum of ten peaks (including five major peaks), and a minimum signal-to-noise ratio of 3.

The resultant .mgf files were searched against the SwissProt database using Mascot 2.5.1 (www.matrixscience.com^46^), with trypsin as the specified enzyme, allowing for two missed cleavages. Carbamidomethylation (C) was chosen as the fixed modification (mass shift = +57.02 Da), and deamidation (NQ; mass shift = +0.98 Da), oxidation (M), oxidation (P), oxidation (K) (mass shift = +15.99 Da; equivalent mass to the process of hydroxylation), and carbamylation (K; mass shift = +43.00 Da) were chosen as variable modifications. The peptide mass tolerance was set at ±10 ppm, and the MS/MS fragment ion mass tolerance was set at 0.5 Da.

For the pots in which bird or fish tissues were cooked, we also searched against a custom database prepared by identifying the common types of proteins identified in SwissProt, and the sequences of the proteins thus obtained from SwissProt were searched using standard protein blast to obtain the sequences of the hits (a maximum of 100 hits). The following proteins searched against different families were considered in creating the custom database (3057 sequences in total):

1. Myosin (9, 11, 1B and heavy chain skeletal muscle), tropomyosin (alpha 1, beta), troponin (C skeletal muscle, I fast skeletal muscle and cardiac muscle, T fast skeletal muscle isoform, cardiac muscle isoform), creatine kinase (B type, M type, S type, U type), collagen (alpha 1(I), alpha 1(II), alpha 2(I)), actin (aortic smooth muscle, alpha cardiac muscle I, alpha skeletal muscle) (all belonging to *Gallus gallus*) and hemoglobin subunits alpha and beta (*Anas platyrhynchos*, *G. gallus*) were all blasted against the Anatidae family.
2. Myosin (9, 11, 1B and heavy chain skeletal muscle), tropomyosin (alpha 1, beta), troponin (C skeletal muscle, I fast skeletal muscle and cardiac muscle, T fast skeletal muscle isoform, cardiac muscle isoform), creatine kinase (B type, M type, S type, U type), collagen (alpha 1(I), alpha 1(II), alpha 2(I)), actin (aortic smooth muscle, alpha cardiac muscle I, alpha skeletal muscle) (all belonging to *G. gallus*), and hemoglobin subunits alpha and beta (*A. platyrhynchos*, *G. gallus*) were all blasted against the Phasianidae family (excluding the genus *Gallus*).
3. Parvalbumin (alpha, beta), unconventional myosin 6 (all *E. lucius*), tropomyosin alpha 1 (*Liza aurata*), glyceraldehyde-3-phosphatase dehydrogenase (*Danio rerio*), hemoglobin (alpha, beta-A/B), beta-enolase, fructose bisphosphate aldolase A (all *Salmo salar*), alpha enolase (*Thunnus albacares*), collagen alpha 2 (I) (*Oncorhynchus mykiss*), actin alpha skeletal muscle, and myosin heavy chain fast skeletal muscle (*Cyprinus carpio*) were all blasted against Esocidae family.
4. Parvalbumin (alpha, beta), actin alpha skeletal muscle, myosin heavy chain fast skeletal muscle (*C. carpio*), glyceraldehyde-3-phosphatase dehydrogenase (*C. carpio* and *D. rerio*), hemoglobin (alpha, beta-A/B), tropomyosin alpha 1 (*D. rerio*), beta-enolase, fructose bisphosphate aldolase A (all *S. salar*), alpha-enolase (*Thunnus albacares*), and collagen alpha 2(I) (*O. mykiss*) were all blasted against Cyprinidae family.

For the Phasianidae family, we excluded the genus *Gallus* as its inclusion would have led to *G. gallus* as one of the species in the database, and because of its proteome diversity, most of the peptides would have matched to it as in the case of SwissProt, thereby depriving us of vital information about whether the use of a more specific database led to better species identification.

For the analysis of the Mascot search results, only the peptides with ion score beyond the identity or extensive homology threshold were considered. For a protein to be considered, there needed to be at least two peptide sequences, with at least one peptide marked in bold red (indicating that the peptide is the highest scoring match for a given MS/MS spectra and that it is the highest scoring protein in which that specific system appears).

The species of the proteins used for obtaining the sequences from UniProt was chosen based on the availability of accurately annotated proteins available in the UniProt database. The custom database has been made available in the Supporting Information.

Proteome Discoverer 2.3 (Thermo Fisher Scientific, UK) was used for label-free quantification to estimate the relative amounts of various proteins extracted and identified in the ceramics, using the Minora algorithm for label-free quantification of proteins. Sequest was used as the database searching step in the Processing workflow (parameters as described above), and a minimum of two peptides were specified in the protein filter stage in the consensus workflow, with other setting maintained at default.

The mass spectrometry proteomics data have been deposited to the ProteomeXchange Consortium via the PRIDE^47^ partner repository with the data set identifiers PXD02899, PXD029035, and PXD027720.

## Results and Discussion

### Absorption of Various Proteins and Their Subsequent Extraction

Mascot search results indicated that cereals were identified at least as conclusively as meat products in all the cases in which a mixture of cereals, meat, and milk was cooked in the ceramic vessels (Table 2) despite the significantly lower amount of protein in cereals as compared to meat and the equal amounts of cereal and meat by mass used in this experiment. Interestingly, milk proteins were rarely identified, despite milk having a comparable amount of protein to cereal grains. Although this is potentially of archeological significance, it may also be due to the milk being cooked with cereals, which are absorbing the milk, resulting in insufficient contact between milk and the ceramic surfaces.

**Table 2:** Number of Proteins from Various Food Products Extracted and Identified from the Six Pots Using the Two Extraction Methods^a^

	DES			GuHCl		
sample	no. of meat proteins	no. of cereal proteins	no. of milk proteins	no. of meat proteins	no. of cereal proteins	no. of milk proteins
A1	**9 (4)**	**7**	0	1 (1)	0	0
A2	**5 (3)**	**3**	0	0	0	0
A3	**4 (1)**	**3**	0	0	0	0
B1	**6 (2)**	**3**	1	2 (1)	1	1
B2	**1**	**3**	0	0	0	0
B3	**1**	**2**	0	0	1	0
C1	**3 (1)**	**2**	0	0	0	0
C2	0	**3**	0	**1 (1)**	0	0
C3	0	**4**	0	0	0	0
D1	2	**3**	0	**3 (1)**	1	**3**
D2	**3 (3)**	**4**	0	1	0	0
D3	**4 (4)**	**4**	**1**	0	0	0
E1	3 (3)	3	0	**5 (2)**	**4**	**2**
E2	4 (2)	4	0	**6 (3)**	**5**	0
E3	**6 (4)**	4	0	1 (1)	4	0

a Numbers in parentheses indicate proteins that were also matched to other species like *Mus musculus*, *Homo sapiens*, and *Canis lupus familiaris*, along with *Bos taurus* or *Sus scrofa*. The bold font indicates the extraction method that furnished a greater number of a specific type of protein (meat vs cereals vs milk) from a particular sample.

The primary cereal proteins identified included various proteins involved in protective function against desiccation during embryo development, including late embryogenesis abundant proteins and Em proteins. Additionally, wheat storage proteins belonging to the gluten group were present, with both glutenin and gliadin (identified as avenin-type proteins) being identified. Gluten group proteins (including avenin, glutenin, and hordein) have been previously identified in archeological samples, along with serpin, purothionin, alpha-amylase inhibitors, and lipid transfer proteins,^7,8^ showing that gluten proteins are potentially suitable as relevant biomarker proteins for cereal processing. Late embryogenesis abundant proteins and Em proteins were the ones most commonly observed in our samples, a pattern not previously observed in archeological ceramics (a list of all the meat, milk, and cereal proteins identified is available as Supplementary Table S4).

Collagen was the most commonly observed meat-derived protein in the pots in which a mixture of meat, cereals, and milk was cooked, along with actin, tropomyosin, and ATP synthase protein in a lesser number of samples. Myosin, which was the most commonly observed protein in the pots in which bird and fish were cooked, was not observed in the pots in which a mixture of cereals, meat, and milk were cooked. However, actin, tropomyosin, collagen, and ATP synthase were observed in the pots in which bird and fish were cooked. Our results suggest that collagen and myosin are potentially the most suitable proteins to use as biomarkers for animal processing, something that was expected as they are among the most abundant proteins in muscle.

Milk proteins were rarely observed despite milk containing a comparable amount of proteins as some of the cereals (2.7 g of protein per 100 g in pearl barley, as compared to 3.5 g of protein per 100 mL for milk). Only four of the 15 samples (B1 using both GuHCl and DES, D1 and E1 using GuHCl, and D3 using DES; three triplicates each of five pots) analyzed furnished milk proteins, with casein being the most common one, along with one instance of beta-lactoglobulin and butyrophillin, all of which have been previously observed in archeological ceramics.^7,8^

**Figure 2:**
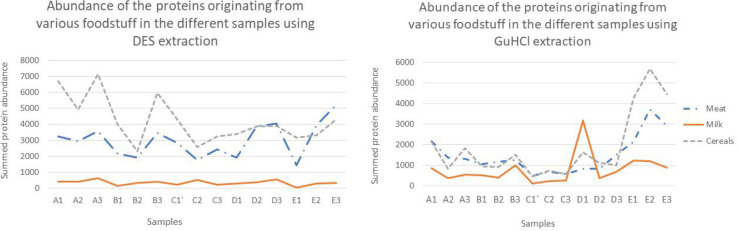
Abundance of the various proteins from meat, cereals, and milk as extracted by the two extraction methods.

Proteins (apart from keratin) identified as belonging to *Bos taurus* and *Sus scrofa* were identified as originating from meat; all proteins belonging to *Hordeum vulgare* and *Triticum aestivum* were identified as originating from cereals, and the various milk-specific proteins (irrespective of the species to which they were identified) were identified as originating from milk (Figure 2; see Supplementary Table S5 for the Proteome Discoverer Protein Report).

Our results showed that cereals, despite having a lower amount of proteins as compared to meat, showed similar protein abundances as compared to meat, and in some cases where a DES extraction was used, substantially higher. This finding has important implications for archeological applications, experimentally supporting the commonly held hypothesis that proteomics techniques, unlike lipidomics, will not discriminate against plant products like cereals in favor of animal-derived products. Although the abundances of the milk proteins observed were, in general, lower as compared to cereals and meat, the Proteome Discoverer results did show milk proteins in most of the samples, unlike the Mascot search results, where milk proteins were observed in only four of the samples after employing our threshold scores.

### Comparison of GuHCl and DES in Extraction of Proteins Absorbed in Ceramics

**Figure 3:**
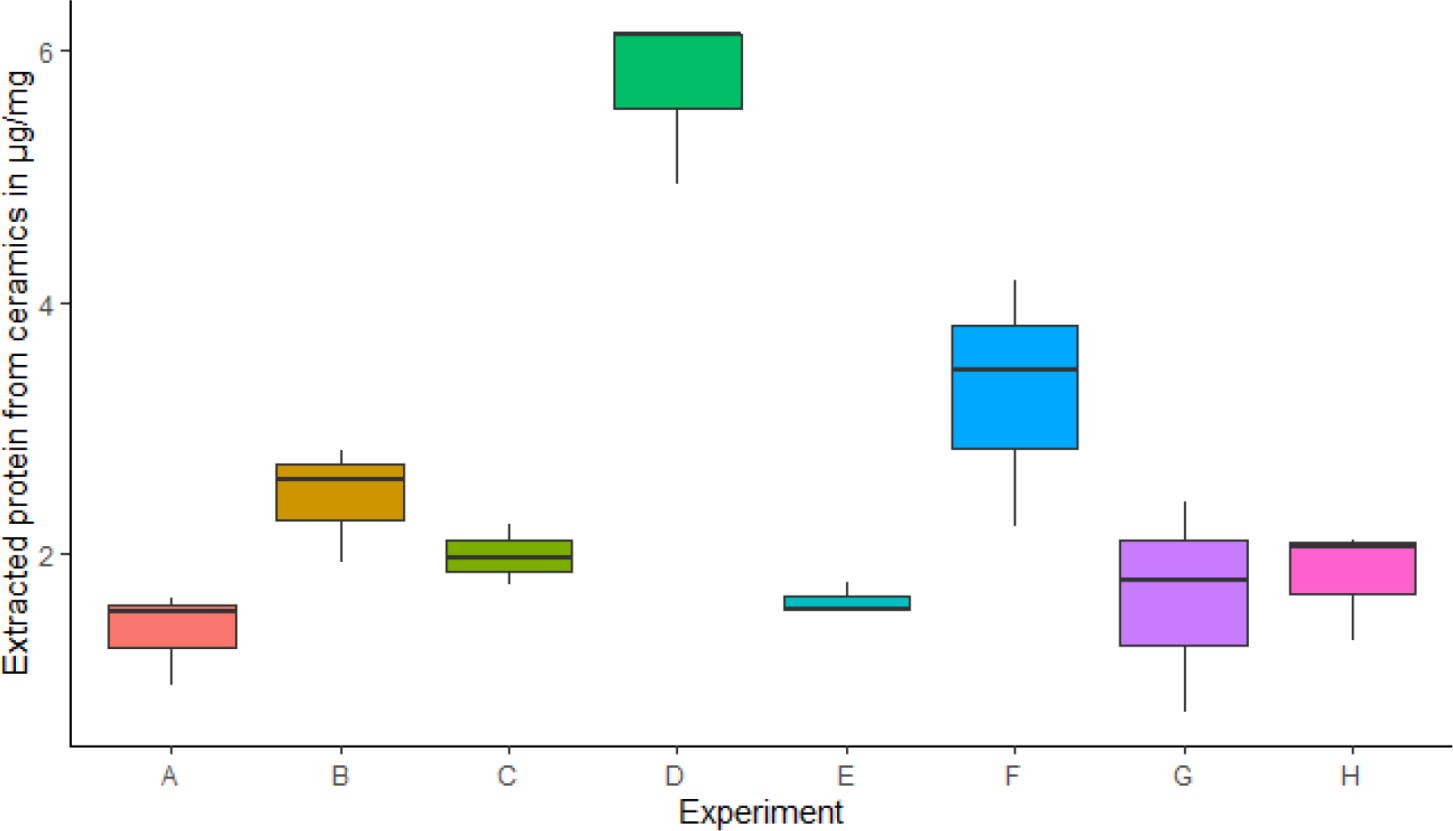
Box plots showing the amount of BSA extracted (in μg mg^–1^) using the various extraction techniques. The various extraction codes in the *x*-axis are as follows: (A) 6 M GuHCl, 4 h, 65 °C; (B) urea–GuHCl DES, 4 h, 65 °C; (C) 6 M GuHCl, 66 h, 4 °C; (D) urea–GuHCl DES, 4 h, 65 °C, ultrasonication; (E) 6 M GuHCl, 4 h, 65 °C, ultrasonication; (F) urea–GuHCl DES, 8 h, 65 °C, ultrasonication; (G) urea–GuHCl DES, 6 h, 65 °C, ultrasonication; (H) urea–GuHCl DES, 2 h, 65 °C, ultrasonication.

For BSA-spiked ceramics, the concentration results from the Cubit measurement were converted into amount of BSA in μg mg^–1^ of ceramics (Supplementary Table S3). The results thus obtained showed that urea–GuHCl DES is more effective at extraction of proteins from the ceramics than 6 M GuHCl under similar conditions. For 6 M GuHCl solution, 66 h at 4 °C was found to be the most effective among the conditions tested, and for urea–GuHCl DES, ultrasonication for 4 h at 65 °C was found to be the most effective (Figure 3). The blank measurements in all the experiments were too low be measured by Qubit at <1 μg/mg.

Once ultrasonication-assisted extraction at at 65 °C for 4 h and incubation at 4 °C for 66 h were established as the most efficient extraction technologies for DES and GuHCl respectively, we analyzed the two sets of experimental ceramics described previously to better compare the performances of GuHCl and the DES as extraction reagents as described in the Experimental Section.

In order to compare the efficiency of the two extraction methods using pots in which beef, pork, cow milk, and cereals were cooked, we used the Mascot search results to identify the number of proteins identified to each of the species (Table 2, Supplementary Table S4). This approach was considered appropriate since cow (*B. taurus*), pig (*S. scrofa*), wheat (*T. aestivum*), and barley (*H. vulgare*) have relatively well-characterized proteomes in the database, allowing for identification of proteins with high certainty. In a majority of the samples, the DES provided a greater number of identifiable proteins and, consequently, a greater proteome diversity (with pot E a notable exception).

**Figure 4:**
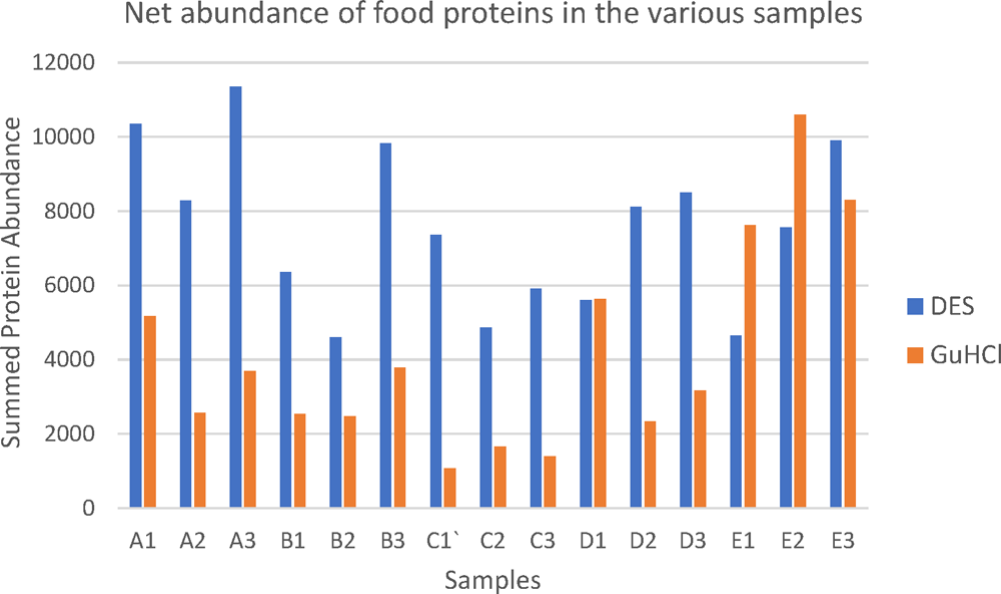
Total abundance of all the food-derived proteins in the various samples as extracted by the DES and GuHCl-based extraction methods.

The amount of proteins extracted using the DES- and GuHCl-based extraction methods was further estimated using the label-free quantification of Proteome Discoverer 2.3 as specified before. The abundance values of the various food proteins (all proteins identified as belonging to *B. taurus*, *S. scrofa*, *H. vulgare*, and *T. aestivum*, and two proteins identified as belonging to beta-lactoglobulin and beta-casein of *Ovis aries*) were summed to provide the net abundance of food proteins in each of the samples. As with the previous approach involving counting the number of proteins, the DES, in general, furnished a greater amount of proteins as compared to the GuHCl-based extraction method, with samples E1 and E2 being the major exception (Figure 4; Supplementary Table S5).

**Figure 5:**
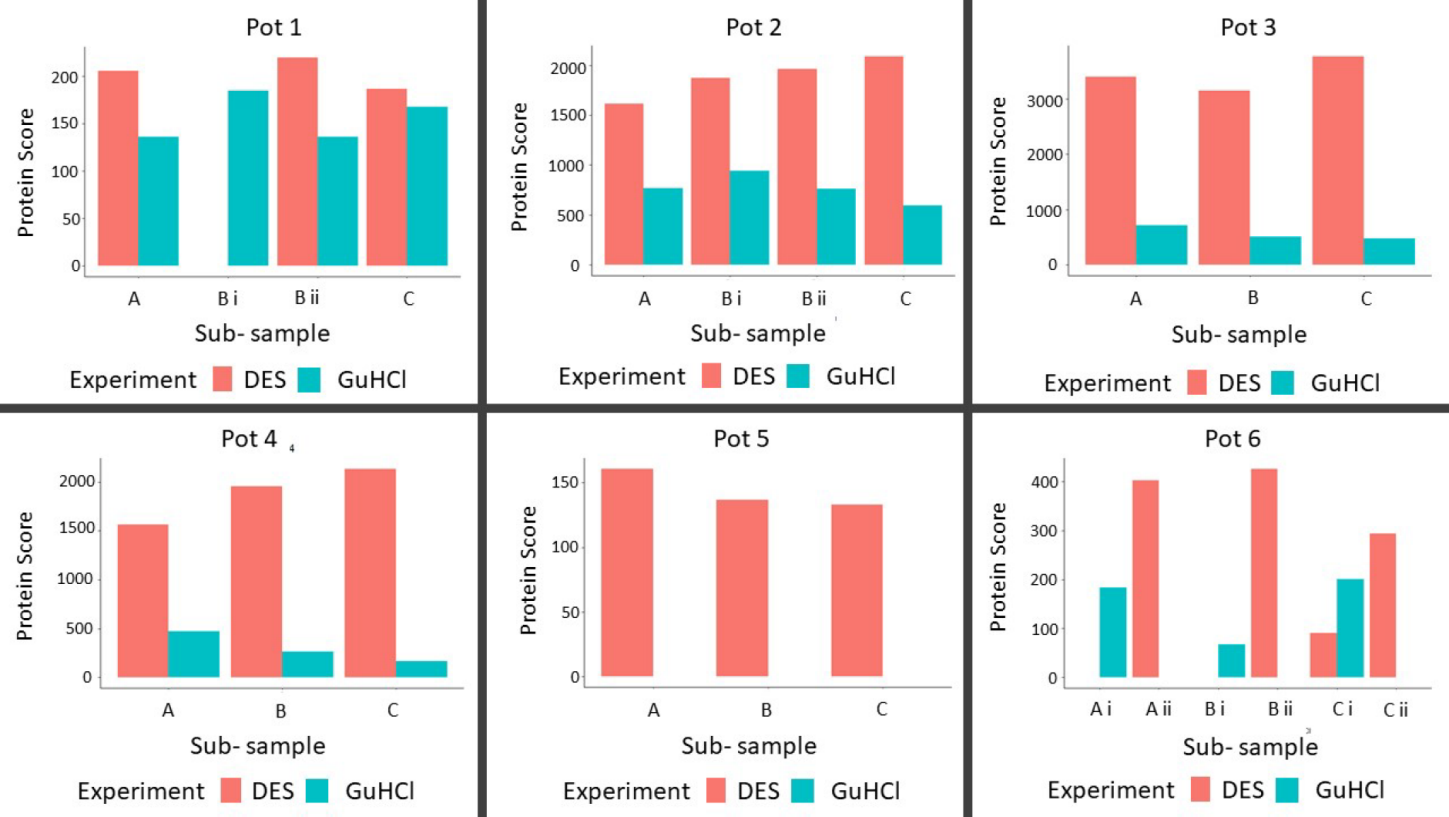
Bar charts comparing the protein scores of the proteins with the highest Mascot score in the six samples (each in triplicate) with SwissProt as the reference database. i and ii indicate that different proteins had the highest score in the GuHCl and the DES extraction. A, B, and C indicate the three subsamples sampled from each cooking pot.

To further compare the efficiency of the two extraction methods, we applied both methods to ceramic pots in which bird and fish were cooked. We chose the highest scoring proteins from each ceramic samples (ignoring all common laboratory contaminant proteins, proteins belonging to *H. sapiens*, and all bacterial or fungal proteins) as per the results obtained by searching against both SwissProt and the custom database and compared their Mascot protein scores (Tables 3 and 4; see Supplementary Tables S6, S7, S9, and S10 for a list of all identified proteins). For samples when the highest scoring proteins were different between the DES and GuHCl extraction, the proteins with the highest score in GuHCl and DES extraction were marked as i and ii, respectively, and the protein scores corresponding to both the extraction methods were plotted (Figure 5, Supplementary Figure S2). We also compared the percentage sequence coverage of the highest scoring proteins extracted using both DES and GuHCl, choosing the proteins as described above (Supplementary Figure S3). Our results showed that with both SwissProt and the custom database, DES, in general, provided a higher protein score as well as higher percentage sequence coverage of the proteins with the highest score in the case of SwissProt (pot 1 being an exception in all the matrices considered, and sample 2 being an exception in measuring the sequence coverage). Of particular interest was pot 5, for which no proteins were identified in any of the triplicates extracted using GuHCl but a small number of proteins in were identified in the triplicates extracted using DES. Given that most of the archeological ceramics are likely to have a limited number of peptides, DES has the potential to be advantageous as compared to GuHCl by being able to extract additional peptides that cannot be extracted by GuHCl solution. Although pot 6 also had a higher sequence coverage (and higher score in some of the triplicates) for the protein with the highest score, DES provided a significantly greater number of peptides (and proteins) in all triplicates from pot 6.

**Table 3:** Species with the Highest Protein Score Identified in the Samples, as per the Search against the SwissProt Database^a^

	species with highest protein scores		other possible common food species in relevant geographical area	
sample	DES	GuHCl	DES	GuHCL
1A	*E. lucius* (PV-alpha, 7) [206]	*E. lucius* (PV-alpha, 2) [136]	*L. aurata*, *C. carpio*	*G. gallus*, *C. carpio*
1B	*E. lucius* (PV-alpha, 5) [220]	*Anguilla anguilla* (TNNC, 2) [185]	*L. aurata*, *C. carpio*	*L. aurata*, *Salmo salar*, *E. lucius*, *C. carpio*, *G. gallus*, *Ctenopharyngodon idella*
1C	*E. lucius* (PV-alpha, 3) [187]	*E. lucius* (PV-alpha, 5) [168]	*C. carpio*	*S. salar*, *O. mykiss*
2A	*G. gallus* (MY1B, 34) [1614]	*G. gallus* (MYH1B, 22) [766]	*B. taurus*, *Argopecten irradians*, *Oryctolagus cuniculus*	*S. scrofa*, *B. taurus*, *A. platyrhynchos*, *Columba livia*
2B	*G. gallus* MYH1B, 42) [1964]	*G. gallus* (MYHC skeletal, 26) [940]	*B. taurus*, *S. scrofa*	*B. taurus*, *O. cuniculus*, *A. platyrhynchos*
2C	*G. gallus* (MYHC, 44) [2090]	*G. gallus* (MYHC skeletal, 20) [597]	*S. scrofa*, *C. carpio*, *O. cuniculus*, *A. irradians*	*B. taurus*, *C. livia*, *A. platyrhynchos*, *O. cuniculus*, *Phasinus colchicus*
3A	*C. carpio* (MYHC skeletal, 53) [3393]	*C. carpio* (MYHC skeletal, 12) [721]	*G. gallus*, *Liza ramada*, *S. salar*	*G. gallus*, *S. salar*
3B	*C. carpio* (MYHC, skeletal, 53) [3152]	*C. carpio* (MYHC skeletal, 14) [508]	*L. ramada*, *S. salar*	*S. salar*
3C	*C. carpio* (MYHC skeletal, 62) [3768]	*C. carpio* (MYHC skeletal, 9) [481]	*G. gallus*, *L. ramada*, *O. mykiss*	*Takifugu rubripres*, *Scomber japonicus*
4A	*C. carpio* (MYHC skeletal, 40) [1561]	*C. carpio* (MYHC skeletal, 16) [474]	*G. gallus*, *Squalis cephalus*, *L. ramada*, *S. salar*	*S. salar*, *T. rubripres*, *S. cephalus*, *G. gallus*, *B. taurus*, *L. ramada*, *O. cuniculus*
4B	*C. carpio* (MYHC skeletal, 45) [1955]	*C. carpio* (MYHC skeletal, 9) [265]	*G. gallus*, *L. ramada*, *S. cephalus*	*S. cephalus*, *S. salar*
4C	*C. carpio* (MYHC skeletal, 48) [2136]	*C. carpio* (MYHC skeletal, 4) [165]	*G. gallus*, *L. aurata*, *S. salar*, *S. cephalus*, *O. mykiss*, *Arctogadus glacialis*	
5A	*G. gallus* (MYH1B, 5) [161]	b		
5B	*G. gallus* (MY1B, 5) [137]	b	*A. irradians*	
5C	*S. scrofa* (MYHC, 4) [143]; *G. gallus* (MYHC skeletal, 4) [133]	b		
6A	*O. cuniculus* (MY4, 10) [410]; *G. gallus* (MYH1B, 10) [402]	*G. gallus* (MYHC skeletal, 8) [183]	*A. platyrhynchos*	
6B	*G. gallus* (MYH1B, 11) [426]	*A. platyrhynchos*, *Aythya fuligula*, and others (HBB, 2) [68]	*G. gallus*, *A. platyrhynchos*	
6C	*G. gallus* (MYH1B,5) [294]	*G. gallus* (TNNC, 2) [201]	*A. platyrhynchos*	

a The number within parentheses indicates the number of sequences, along with the identified protein. The number in square brackets indicate the protein score.

b Not applicable.

**Table 4:** Species with the Highest Protein Score, as per Search against the Custom Database^a^

	species with highest protein scores		other possible common food species in relevant geographical area	
sample	DES	GuHCl	DES	GuHCL
1A	*E. lucius* (COL1A2, 12) [560]	*E. lucius* (COL1A1, 21) [806]		*Coturnix japonica*, *C. carpio*
1B	*E. lucius* (TA1X1, 16) [701]	*E. lucius* (COL1A1, 24) [785]	*C. carpio*	*A. platyrhynchos*, *Cygnus atratus*, *P. colchicus*
1C	*E. lucius* (TA1X1, 10) [479]	*E. lucius* (COL1A1, 22) [1102]		*A. platyrhynchos*, *A. fuligula*
2A	*A. platyrhynchos* (MYHC_skeletal, 64) [4515] (*M. gallopavo*)	*Coturnix japonica* (MYHC_skeletal, 67) [2625]	*C. japonica*, *P. colchicus*, *A. fuligula*, *E. lucius*	*A. platyrhynchos*, *P. colchicus*, *C. carpio*, *E. lucius*, *A. fuligula*, *Anser cygnoides domesticus*
2B	*A. platyrhynchos* (MYHC_skeletal, 84) [5700]	*A. platyrhynchos* (MYHC_skeletal, 54) [2404]	*C. japonica*, *P. colchicus*, *A. fuligula*, *E. lucius*, *C. carpio*, *A. cygnoides domesticus*, *Anser anser*	*P. colchicus*, *C. japonica*, *A. fuligula*, *E. lucius*, *C. carpio*
2C	*C. japonica* (MYHC_skeletal, 79) [5695]	*P. colchicus* (MYHC_skeletal, 43) [1644]	*A. platyrhynchos*, *A. fuligula*, *P. colchicus*, *E. lucius*, *C. carpio*, *A. cygnoides domesticus*	*C. japonica*, *A. platyrhynchos*, *E. lucius*, *A. fuligula*, *A. cygnoides domesticus*
3A	*C. carpio* (MYHCl, 111) [9745] (*C. auratus*)	*C. carpio* (MYHC, 37) [1594]	*E. lucius*, *A. fuligula*, *C. japonica*, *P. colchicus*, *A. platyrhynchos*	*E. lucius*, *A. platyrhynchos*, *A. fuligula*, *A. cygnoides domesticus*
3B	*C. carpio* (MYHC, 198) [10431] (*C. auratus*)	*C. carpio* (MYHC, 29) [1019] (*C. auratus*)	*C. carpio*, *C. japonica*, *P. colchicus*, *A. platyrhynchos*	*A. platyrhynchos*, *A. fuligula*, *A. cygnoides domesticus*, *E. lucius*
3C	*C. carpio* (MYHC, 112) [10301] (*C. auratus*)	*E. lucius* (MYHC, 16) [555] (*C. auratus*)	*E. lucius*, *A. platyrhynchos*, *A. fuligula*, *P. colchicus*	*C. carpio*
4A	*C. carpio* (MYHC_embryonic, 91) [5583] (*C. auratus*)	*C. carpio* (MYHC_embryonic, 47) [1937] (*C. auratus*)	*E. lucius*, *A. fuligula*, *C. japonica*, *A. cygnoides domesticus*, *A. platyrhynchos*	*E. lucius*, *A. fuligula*, *C. japonica*
4B	*C. carpio* (MYHC_embryonic, 106) [7284] (*C. auratus*)	*C. carpio* (MYHC_embryonic_2, 27) [912] (*C. auratus*)	*E. lucius*, *A. fuligula*, *C. japonica*, *Anser anser*, *A. platyrhynchos*	*E. lucius*, *C. japonica*, *A. fuligula*
4C	*C. carpio* (MYYHC_embryonic, 97) [7065] (*C. auratus*)	*C. carpio* (MYHC_embryonic, 16) [587] (*C. auratus*)	*E. lucius*, *A. fuligula*, *A. anser*, *P. colchicus*, *A. platyrhynchos*	*E. lucius*, *A. fuligula*
5A	*A. platyrhynchos* (MYHC_skeletal_X1, 16) [422] (*C. atratus*)	no identified proteins	*A. platyrhynchos*, *A. cygnoides domesticus*	
5B	*A. platyrhynchos* (TAlpha, 14) [429] (*Oxyura jamaicensis)*	no identified proteins	*C. japonica*, *P. colchicus*, *A. fuligula*, *A. platyrhynchos*	
5C	*C. japonica* (TBeta_X14, 10) [412]	no identified proteins	*A. cygnoides domesticus*, *A. platyrhynchos*, *A. fuligula*, *P. colchicus*	
6A	*A. anser* (MYHC1, 24) [1048]	*A. platyrhynchos* and others (MYHC_skeletal, 14) [446]	*A. platyrhynchos*, *A. cygnoides domesticus*, *C. japonica*, *P. colchicus*, *A. fuligula*, *E. lucius*	*A. fuligula*, *Mareca penelope*
6B	*A. platyrhynchos* (MYHC_skeletal, 23) [1137]	*A. platyrhynchos* and multiple others (HBB, 4) [112] (*M. gallopavo*)	*A. fuligula*, *C. japonica*, *A. cygnoides domesticus*, *P. colchicus*	*M. penelope* (multiple other Anatidae hemoglobin)
6C	*A. platyrhynchos* and multiple others (MYHC_skeletal, 14) [744]	*(A. platyrhynchos* and multiple others) (TNNC protein, 2) [236]	*A. platyrhynchos*, *A. fuligula*, *M. penelope* (hemoglobin matched to multiple other Anatidae)	

a The number within parentheses indicates the number of sequences, along with the identified protein. The number in square brackets indicate the protein scores. When the species is within parentheses, it indicates that those species had the highest protein scores but were not present in the geographical area under consideration.

A similar trend between the DES and GuHCl extraction was observed when we estimated the abundance of the proteins with the highest Mascot score (as described above) using the label-free quantification in Proteome Discoverer 2.3 (Thermo Fisher Scientific, UK). The default LFQ processing and consensus workflow was used, with search parameters described as above and a minimum of two peptides required in the peptide and protein filter stage in the consensus workflow. In general, DES in general furnished greater abundances of the proteins identified as compared to the GuHCl-based method (apart from pot 1 and one subsample of pot 5; Supplementary Figure S4, Supplementary Table S8). Surprisingly, for pot 5A, in which no proteins of interest were identified using GuHCl once we used our Mascot cutoff criteria (score cutoff, as well the presence of peptides marked in bold red), GuHCl furnished a greater abundance of the protein under consideration as compared to the DES extraction.

**Figure 6:**
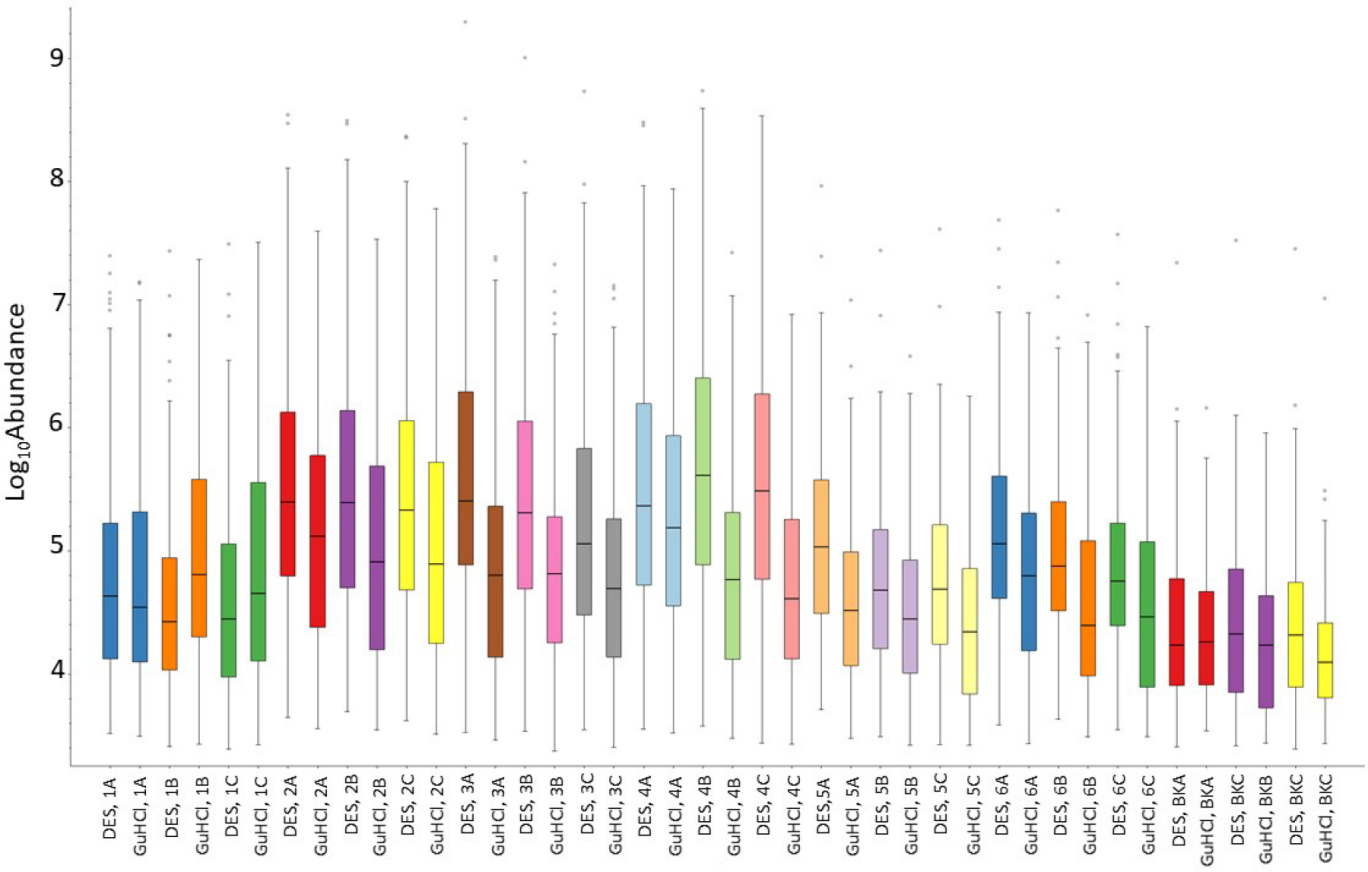
Net protein abundance (non-normalized, expressed as log 10 abundance) in the various samples determined using Proteome Discoverer with the custom database as the reference database.

To further compare the amount of protein extracted by the GuHCl- and DES-based methods, we searched the files against the custom database as described before and measured the net protein abundance using label-free quantification techniques available in Proteome Discoverer (version 2.3, Thermo Fisher Scientific, UK). The default processing and consensus workflow templates for Precursor Quantification and LFQ were used with a minimum of two peptides specified in the peptide and protein filter stage of the consensus workflow. The non-normalized protein abundances corresponding to all the samples were plotted as log 10 abundance (Figure 6; see Supplementary Table S11 for the Proteome Discoverer results statistics). The results further confirmed the improved efficacy of DES as compared to GuHCl, with the DES furnishing a greater abundance of proteins in a majority of the samples (with the exception of samples 1B and 1C).

We also counted the total number of peptides with score above the identity and homology threshold as identified using Mascot, and from it subtracted the number of peptides identified in the decoy database (Supplementary Table S12) to use the resultant quantity as another proxy for the extraction efficiency of the two methods. Using the number of peptides above the identity threshold, we constructed Bland–Altman plots (Supplementary Figure S5), which are commonly used to compare a reference method to a newly developed one. From the Bland–Altman analysis, the mean difference (bias) was found to be −282 (+163.5632); a negative value indicating that the established method (GuHCl in this case), on an average, furnished a lower number of peptides than the novel method (the DES-based method one). Although we acknowledge the limitations of this comparison as the number of peptides recovered from the samples differed widely, it nevertheless provides further supporting evidence of the superiority of DES as an extraction agent. This was further supported by individual comparisons involving the number of peptides above the identity and homology threshold; irrespective of whether we considered those peptides with a score above the identity or homology threshold, DES provided, on average, a greater number of peptides in four of the six samples (Supplementary Figure S6, with pot 1 the exception).

### Influence of Database Choice in Identification of Species by Shotgun Proteomics

To determine the confidence with which proteomics can help achieve species-level identification, the pots in which duck and fish were cooked were analyzed for absorbed proteins, with the identified species with the highest protein score (from searching the .mgf files against the SwissProt database) tabulated (Table 3). For this tabulation purpose, only the species that are plausible as common food sources and present in the geographical area under consideration were considered from among all the proteins identified in Mascot search. All common laboratory proteins, bacterial and fungal proteins, and all proteins belonging to *Rattus*, *Mus*, and *H. sapiens* were ignored. Apart from the proteins mentioned in Table 3, additional proteins (including muscle proteins such as myosin and tropomyosin) belonging to several other species like dogs (*Canis lupus familiaris*), goldfish (*Carassius auratus*), and zebrafish (*D. rerio*), along with proteins belonging to organisms not present in the geographical area under consideration like wild turkey (*Meleagris gallopavo*), African clawed frog (*Xenopus laevis*), and Andean goose (*Chloephaga melanoptera*) were identified in the pots among others (see Supplementary Tables S6 and S7 for a list of all the proteins identified using SwissProt).

For all samples, the species with the highest protein score accurately matched with whether bird or fish was cooked in the pot, but more specific identification to the species level was not possible. Northern pike (*E. lucius*) was accurately identified as species, but the taxonomic origins of processed avian carcasses and the cyprinid fishes were not. The black grouse (*L. tetrix*) was identified as chicken (*G. gallus*), a species belonging to the family Phasianidae, as was duck (Russian Chirok and northern pintail; family Anatidae). This discrepancy between proteomic identification and what was known to be present brings to focus the inherent limitation of the probability-based sequence matching approach with limited relevant protein sequence availability. For example, when *L. tetrix* was specified as an organism in a UniProt database search, it did not have any proteins in the curated component (i.e., in SwissProt), but *G. gallus* had multiple such proteins. Thus, it is not possible to identify black grouse by proteomics if SwissProt is considered as the database until it is further populated with relevant sequence information and improved with better understanding of sequence variation within identified proteins.

**Figure 7:**
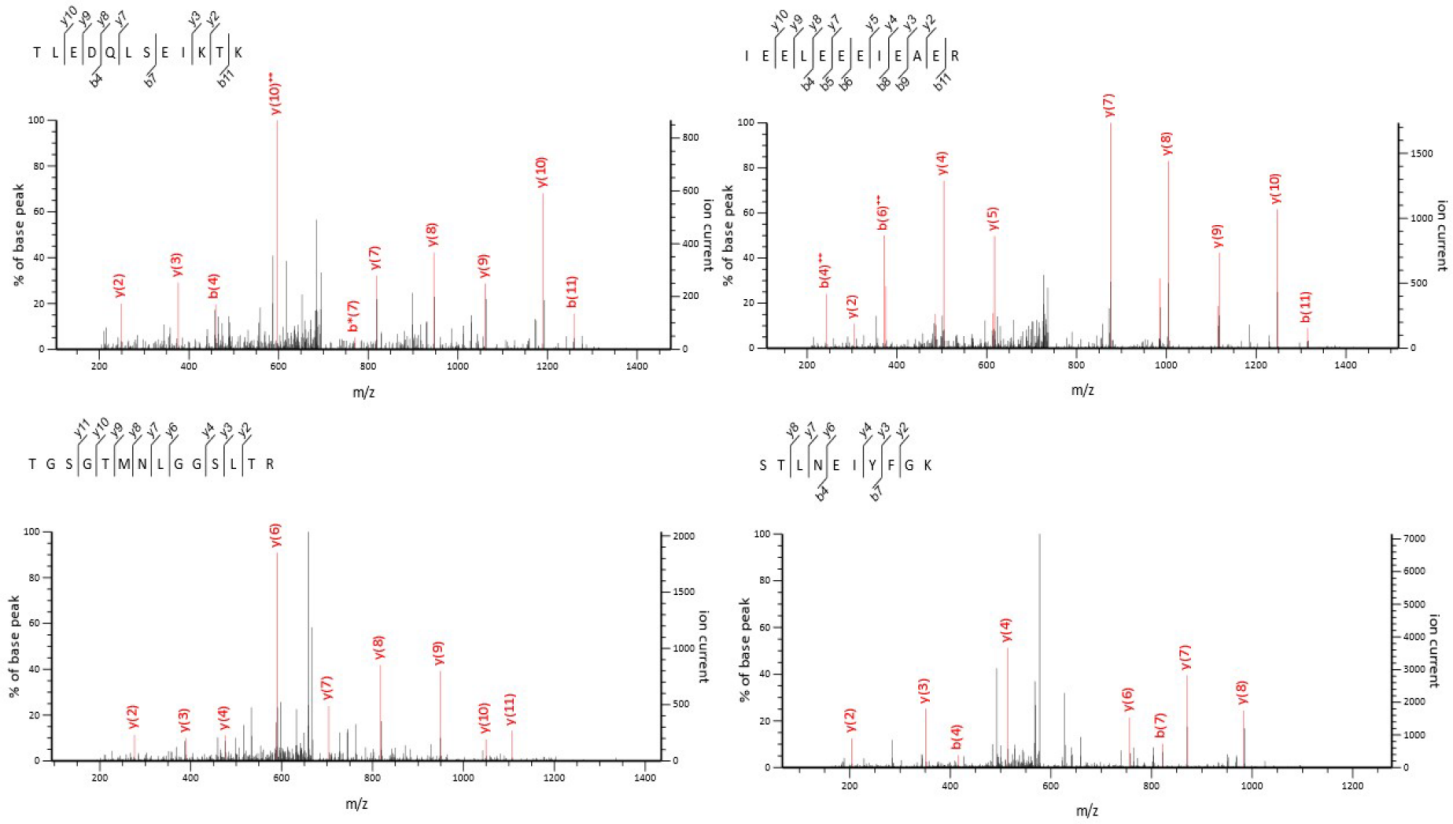
Representative tandem spectra of peptides identified to be from *G. gallus* identified in samples 3A and 4A. The top row contains peptides identified as belonging to myosin heavy chain, skeletal muscle, and the bottom row peptides belonging to F-actin capping protein.

If additional species (in addition to the one with the highest protein score) were considered, a broader range of species could be identified in the samples. One of the sample 2 triplicates in which black grouse was cooked showed proteins not only belonging to other birds (e.g., *C. livia*, *A. platyrhynchos*), but also to rabbit and aquatic animals like common carp (*C. carpio*) and bay scallop (*A. irradians*). This was presumably due the similarities between the various protein sequences across species as well as the inherent limitations of probability-based matching and the proteomic workflow, which is aimed at identifying the proteins present and not necessarily their accurate species. Similarly, samples 3 and 4, in which fish were cooked, matched chicken as one of the identified species (see Figure 7 for representative MS/MS spectra). The MS2 spectra of some peptides were also checked to see if the nature of the MS2 spectra of peptides matched in Mascot to proteins specifically originating from species of animals cooked in the pots differed from peptides matched to other species. In most of the cases of protein matches, there was good coverage of fragment ions originating from a number of peptides, a trend which was also observed when the custom database was used as the reference database (Figure 7, Supplementary Figures S7 and S8), thereby indicating that a quick visual inspection of the nature of fragment ions observed was not sufficient to accurately distinguish between proteins originating from species cooked in the pots and other matches.

This misattribution of species necessitates caution if proteomic studies of pottery residues are used to identify exploitation of a variety of resources, particularly the processing of a secondary resource along with a primary one. For example, it is established that hunter–gatherers and pastoralists inhabiting the Siberian forests and steppes during the Neolithic and Bronze Ages regularly exploited fish,^38,48,49^ but it is unknown to what extent they used additional resources such as waterfowl and forest birds that provide additional seasonal sources of fats and proteins, and it is unlikely that minor contributions from resources can be accurately identified by a proteomic approach alone. Similarly, extreme caution is to be exercised if proteomic data is used to comment on the historic distribution of species, regional extinction phenomenon, and other geographical distribution factors.

However, the proteins identified from SwissProt provided for robust tissue-specific identification, with common constituents of muscle tissues including myosin, parvalbumin, enolase, and hemoglobin being identified, potentially providing relevant information absent in the archeological record about the precise carcass parts processed for food.

To further analyze how analytical decisions to filter proteomic data through a more limited database can affect protein identification, we created a custom database as described previously. Similar to the searches against SwissProt, the proteins with the highest score and other possible proteins from food sources were identified, after taking geographical limitations into consideration (Table 4; for a detailed list of all the proteins identified, see Supplementary Tables S9 and S10).

Interestingly, in sample 2, in which black grouse (*L. tetrix*, a bird from the Phasianidae family) was cooked, a large number of proteins matched to various Anatidae species were also identified. In addition, multiple fish proteins were identified, including proteins belonging to northern pike and common carp. Consequently, it is possible for a large number of matches to arise from multiple closely related species even when the actual species in question is absent from the database (as in the case of *L. tetrix*), even if the target species is from a different family. Thus, the use of shotgun proteomics to identify specific species is fraught with uncertainty; misidentifications can be common unless we are confident that our target species is well represented in the database.

As expected, the use of a smaller and more specific database (the curated database as opposed to SwissProt) led to statistically more robust protein identification, as indicated by the higher protein scores. However, this approach also led to a greater potential for mismatch, where the peptides from a particular species were matched a different species, one which is better represented in the database. *C. auratus* was the species with the highest protein score in two out of the three pots in which fish was cooked, presumably as its proteome is so well-characterized. Similarly, proteins belonging to *Sinocyclocheilus* sp. were identified in a majority of the samples. This appeared to be more common in samples 3 and 4, from which a greater variety of proteome was extracted, than in sample 1, which furnished a less complex proteome (see Supplementary Tables S9 and S10 for a detailed list of all the proteins identified). A similar trend was observed for birds, where *O. jamaicensis* (ruddy duck, North America), *C. atratus* (black swan, Australia), and *M. gallopavo* (turkey, North America) were commonly identified in samples in which duck or black grouse were cooked (see Supplementary Figures S7 and S8 for some representative MS/MS spectra).

Our results demonstrate that in cases when a specific protein belonging to a species was absent in the database, peptides originating from that organism could be matched to related organisms due to the similarity between the sequences of various proteins across different organisms (as in the cases of *L. tetrix* and *A. acuta* in these samples). This indicates that the confidence with which Mascot results can identify the species of the peptides is dependent on the size and evolutionary diversity of the protein under consideration. As such, for confident taxonomic assignment using proteomics, it is essential to devise a way to compare between various proteins belonging to different species in a database, considering the proteome diversity of the respective species present, as well as compare the evolutionary diversity of various proteins. As an example, black grouse (cooked in pot 2) has 89 UniProt entries as of 25th September 2022 (mostly oxidase-reductase type enzymes), with no abundant muscle/bone proteins like myosin or collagen. On the contrary, chicken has 34,988 entries, which ensures that a sequence is much more likely to be matched to chicken than black grouse due to the much greater number of available sequences. Further, this phenomenon of proteins matching to different species also appeared to be protein-dependent; proteins like myosin and actin were more likely to be identified as belonging to a different species than the one from which they were derived as compared to proteins like parvalbumin. We postulate that this is possibly due to the different size and rate of evolution of the different proteins, leading to different degrees of similarity of preserved sequences and therefore molecular diversity across different taxa. For example, the rate of substitution in myosin 1B appears substantially higher in birds (e.g., 68 changes between duck and chicken for a protein almost the same length as both type 1 collagen chains combined) in comparison to collagen (15 changes), but relatively unchanged for mammals (e.g., only 3 substitutions between sheep and goat). As such, relying solely on proteomics to identify exploitation of specific organisms from residues in ceramics can lead to misleading conclusions, resulting in inaccurate information about historic distribution of species and the relative importance of various resources that are exploited. This also leads to potential concerns about using proteomics to differentiate between dependence on terrestrial and aquatic resource use (although our results show the capability to broadly distinguish between fish and birds) and shows the present difficulty in using proteomics to study use of domesticated species vs the use of wild sources. Much care is to be taken if proteomics is utilized to identify species-specific resource use in antiquity, particularly in cases when additional confirmatory sources are absent. Although the present study investigating database effects on species identification considered only birds and fish, we note that these two taxa are on the two extremes of sequence rate change when collagen is considered, birds (*Gallus* and *Anas* species) showing a substitution rate of ∼0.1 amino acid per million years and fishes (*O. mykiss* and *O. keta*) showing a substitution rate ∼2.7 amino acids per million years.^50^ The fact that both birds and fish showed mismatches between the known species and the proteomics-based identification indicates that this is a possibility across other species as well.

As expected, muscle proteins made up the bulk of the proteins identified in most of the samples, with collagen and myosin being the common proteins with highest scores in all the samples irrespective of the database choice. Myosin is the most abundant muscle protein, making ∼25% of all the muscle proteins,^51^ while collagen is the major structural protein in the extracellular matrix of skeletal muscle, making up to 10% of the dry muscle weight.^52,53^ Because of their ubiquitous nature, these proteins make prime candidates for absorption and subsequent preservation in ceramic matrix during cooking. However, both of these proteins are highly conserved in terms of their sequence,^54−58^ making differentiation at species level difficult, although collagen has been shown to achieve this for some^59,60^ but it requires particular peptides. The species level information from residue analysis is thus dependent on three factors. Two of them, the extent of absorption and subsequent survival of dietary proteins and their evolutionary diversity across various species, are dependent on the inherent nature of the protein, while the other one is the taxonomic diversity of the protein under consideration present in the database.

**Figure 8:**
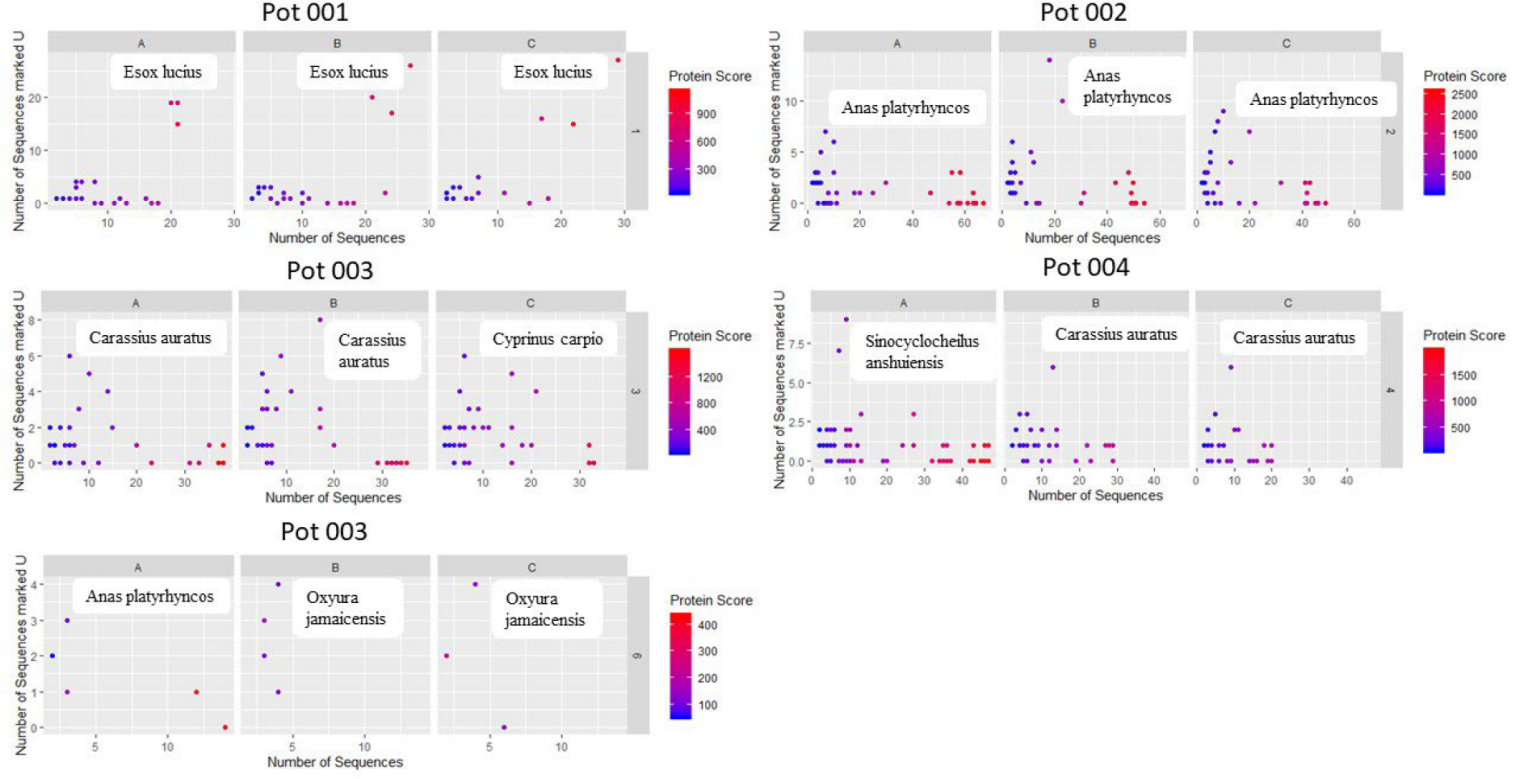
Scatter plots showing the number of sequences marked U (as determined by Mascot) against the total number of sequences. The species of the proteins with the highest number of sequences marked U have been mentioned. Each dot represents a protein (Custom database).

To determine if the number of sequences unique to a particular protein family as determined by Mascot (marked as U) can be used for accurate identification of specific species, we plotted the number of sequences marked as U for all the proteins against the total number of identified sequences for that particular protein (Figure 8, Supplementary Figure S9). When the custom curated database was used, sample 1 (the sample for which the species identifed from Mascot search matched with the known species cooked in the pot) had a number of proteins with the number of sequences marked U comparable to the number of total identified sequences, as well as a high protein score (Figure 8). For the remaining samples, this pattern was not observed.

When SwissProt was used as the database under consideration and similar graphs were plotted, a similar pattern was not obvious (Supplementary Figure S9). For sample 1, *E. lucius* was the species with number of sequences marked U comparable to the number of total identified sequences and the highest protein score. However, sample 3 and sample 4, in which *C. carassius* and *L. idus* were the actual species respectively, showed *C. carpio* as the species with the highest number of peptides marked U.

The moderate number of samples considered as part of this study, while posing some limitations in their interpretation, nevertheless highlights the potential for the use of peptides marked U for accurate identification of species, despite its uncommon use as a productive factor in identification of proteins from archeological ceramics. However, this approach should be used in conjunction with other factors, including a high protein score, identification of multiple proteins, and the presence of a large number of sequences. Although the presence of two peptides have been considered as a minimum threshold for accurate identification of proteins, our results suggest that its presence is not suitable for use as the sole sufficient criteria if accurate species identification is to be achieved.

## Conclusions

Here, we performed a set of cooking simulation experiments in ceramic pots, investigating the absorption and subsequent extraction and identification of various food proteins from the ceramics. Despite cereals containing relatively lower amounts of protein as compared to meat, cereal proteins were identified as readily and at comparable amounts to meat proteins from the ceramic pots. Our results provide experimental verification for the ability of proteomics techniques to identify both plant- and animal-based products when they are processed together. Further, considering the fact that cereal proteins were identified at least as readily as animal proteins, our results further support the commonly held notion that proteomics can be a complementary technique to lipidomic analysis, which can be biased in favor of lipid rich animal-derived resources.

We also developed an improved extraction technique for recovery of absorbed proteins from ceramics, which involves the first reported use of a DES for extraction of ceramic-bound proteins. Our results showed that in most of the cases, use of DES resulted in greater protein recovery. DES also resulted in a quicker extraction process, taking approximately 4 h as compared to the 66 h required for the GuHCl extraction. However, the extraction involving DES was also more sensitive to experimental variations. Because of the higher melting point of the DES (∼58 °C) and its sensitivity to the presence of water,^24^ the mixing of the DES and the ceramics while maintaining the liquid nature of the DES can be challenging, something which is further exacerbated by the viscous nature of the DES as compared to water. This can prevent adequate mixing of the ground ceramics and the extraction media, something which we believe could explain the anomalous results in some samples where DES resulted in lower protein recovery as compared to the GuHCl-based process.

Our analysis of experimental ceramics in which fish and birds were cooked suggests that although accurate species-specific identification of resources from proteomic analysis of residues from ceramic vessels is fraught with uncertainty, it is nevertheless possible to accurately identify the broad patterns of resource use. In the present work, the species with the highest protein score in all the samples analyzed concurred with whether bird or fish were processed in the pot, allowing us to accurately identify avian processing in archeological deposits, something which has been beyond the capability of residue analysis until now. This is particularly important for reconstruction of dietary practices among hunter–gatherers of the Siberian Neolithic. Although aquatic resources played an important role in the Siberian Neolithic, waterfowl and other birds could have augmented the diet of the hunter–gatherers seasonally, something which is difficult to detect by conventional lipid analysis. However, depending on the species involved it is difficult to ascertain by proteomics alone if minor quantities of a secondary resource are used along with a primary one, since our samples in which fish were cooked often showed minor amounts of proteins belonging to birds (duck, chicken, etc.) and *vice versa*.

Our results also suggest that not only are proteomics-based identifications biased in favor of the well-represented organisms in the database as expected, but proteins from many related organisms can be identified as belonging to their more closely represented counterpart. Thus, although proteomics is undoubtedly a versatile and useful part of the archeologists’ arsenal for identifying use of animals and plants commonly used in Western, industrialized economic systems (and hence, particularly well represented in the proteomic database), it is less useful for identifying exploitation of local wild resources or for constructing a timeline of animal and plant domestication; wild species, which are less well-characterized in databases, can be easily misidentified as better-characterized domestic species (for example, the identification of black grouse as chicken).

The reliable use of proteomic residue analysis of ancient ceramic vessels as a means to accurately identify the food resources exploited by humans requires further experimental simulation of cooking and burial processes vital for understanding the absorption, degradation and subsequent identification of various proteins. Simulated burial experiments can play an important role in this regard, allowing us to study various diagenetic processes and to better understand the survival of various proteins, potentially allowing for improved interpretation of proteomic data. The present work identifies some of the limitations to the identification of proteins and their sources, and against the backdrop of ever-improving public databases due to advances in genomic sequencing, further work on more simulation experiments involving various cooking and simulated burial experiments will allow us to better understand resource use and dietary practices in antiquity.

## Supporting Information Available

Figure S1, phase diagram showing the formation of a DES; Figures S2–S4, bar charts showing the Mascot and protein scores (Custom database), sequence coverage, and proteome discoverer abundance of the highest scoring proteins using the two methods; Figure S5, Bland–Altman plot to compare between peptides extracted using GuHCl and DES; Figure S6, bar charts showing the number of peptides extracted using the two methods; Figures S7 and S8, additional examples of tandem mass spectra; and Figure S9, scatter plots showing the number of sequences marked U against the total number of sequences (SwissProt database) (PDF)
Table S1, mass of the various BSA-spiked proteins samples and the amount of BSA extracted; Table S2 and S3, mass of the ceramics sampled from pots in which milk, meat, and cereals were cooked and duck and fish were cooked; Table S4, Mascot search results showing the various food proteins identified from pots used for cooking milk, meat, and cereals; Table S5, Proteome Discoverer results of pots used for cooking milk, meat, and cereals; Tables S6 and S7, Mascot search results of the pots used for cooking duck and fish with SwissProt database using DES and GuHCl; Table S8, Proteome Discoverer results of pots used for cooking milk, meat, and cereals with SwissProt database; Tables S9 and S10, Mascot search results of the pots used for cooking duck and fish with custom database using DES and GuHCl; Table S11, Proteome Discoverer result statistics of the pots used for duck and fish with custom database and grouped on the basis of extraction technique; Table S12, number of peptides identified from the pots used for cooking duck and fish (XLSX)
Custom database curated from proteomic analysis (TXT)
