# Supplementary material for: Novel Deep Eutectic Solvent-Based Protein Extraction Method for Pottery Residues and Archeological Implications
Source: J Proteome Res. 2022 Oct 21;21(11):2619–34. doi: 10.1021/acs.jproteome.2c00340 (PMC9639204; doi:10.1021/acs.jproteome.2c00340)
Supplement: Supplementary file 1 — pr2c00340_si_001.pdf [file pr2c00340_si_001.pdf]

A novel deep eutectic solvent-based protein extraction method for pottery residues and archaeological implications

Manasij Pal Chowdhury[*1,2], Cheryl Makarewicz[3,4], Henny Piezonka[3,4], Michael Buckley[1,2]

[1] Manchester Institute of Biotechnology, University of Manchester, 131 Princess Street, Manchester, M1 7DN, UK

[2] Interdisciplinary Centre for Ancient Life, Department of Earth and Environmental Sciences, University of Manchester, Oxford Road, Manchester, M13 9PL, UK

[3] Institute for Prehistoric and Protohistoric Archaeology, Johanna-Mestorf Strasse 2-6, Kiel University, D-24118, Germany

[4] Cluster of Excellence ROOTS: Social, Environmental, and Cultural Connectivity in Past Societies, Kiel University, Leibniz Strasse 1, Kiel University, 24118, Germany

* Corresponding author

Correspondence: manasij.palchowdhury@manchester.ac.uk

Supplementary_Information_Figures.docx. Contains supplementary figures showing the phase diagram involved in the formation of DES, examples of tandem mass spectra, and additional bar and scatter charts comparing the efficiency of the DES and GuHCl-based methods using protein scores, sequence coverage, and number of peptides.

Supplementary_Table_DES_Paper.xlsx. Contains additional tables showing the various masses of the samples involved in analysis, proteins identified in all the samples using Mascot and Proteome Discoverer, and the Proteome Discoverer results statistics.
database using DES and GuHCl respectively.

Custom_Database.fasta.txt. Contains the curated custom database.

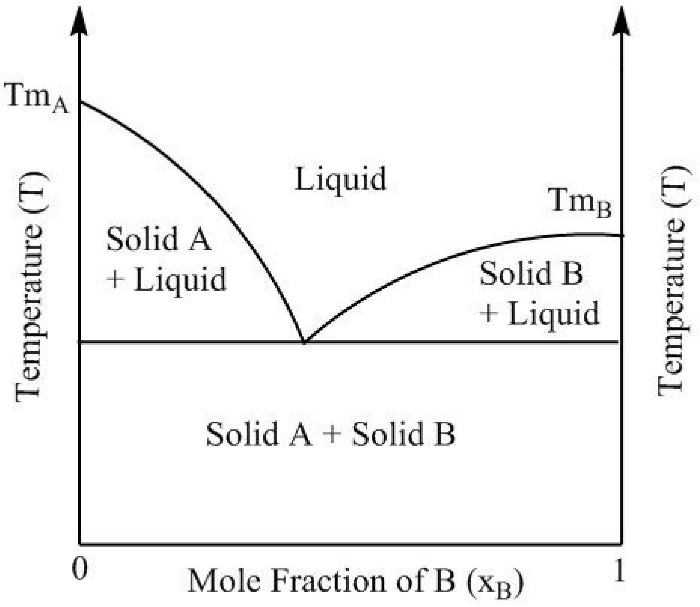

Supplementary Fig. 1: Phase diagram showing the freezing point depression in a eutectic mixture, leading to formation of a DES.

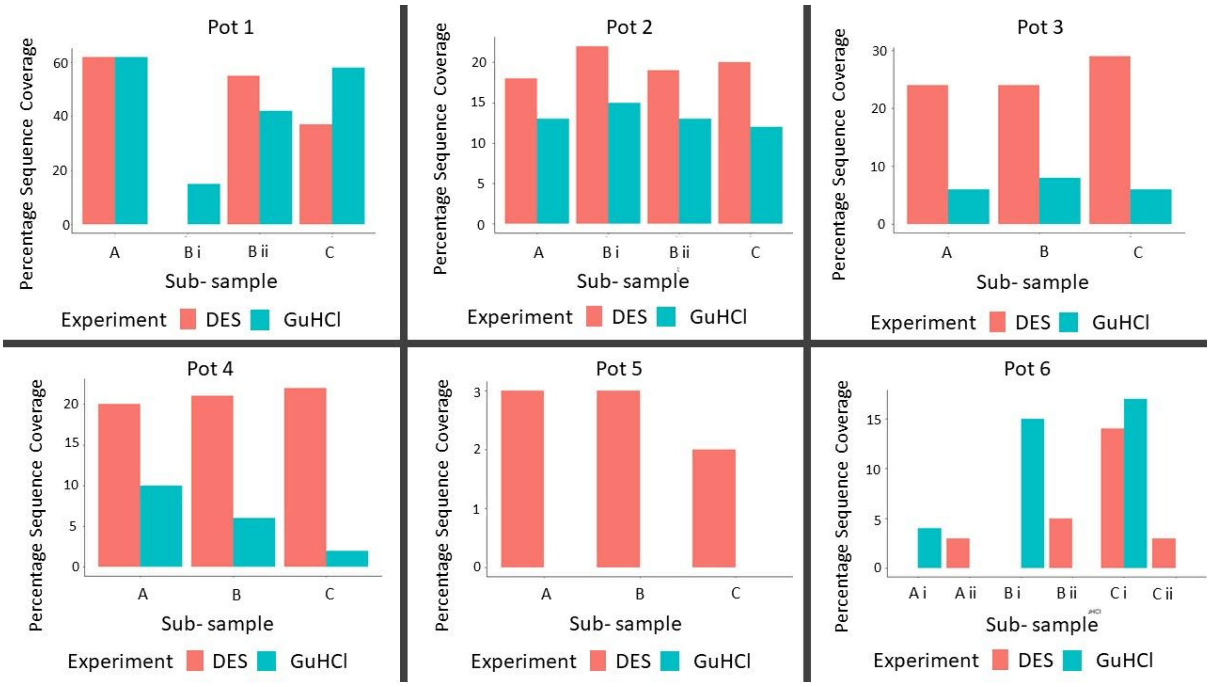

Supplementary Fig. 2: Bar charts comparing the Mascot sequence coverage of the proteins with the highest Mascot score in the six samples (each in triplicates) with SwissProt as the reference database. I and ii indicates that different proteins had the highest score in the GuHCl and the DES extraction.

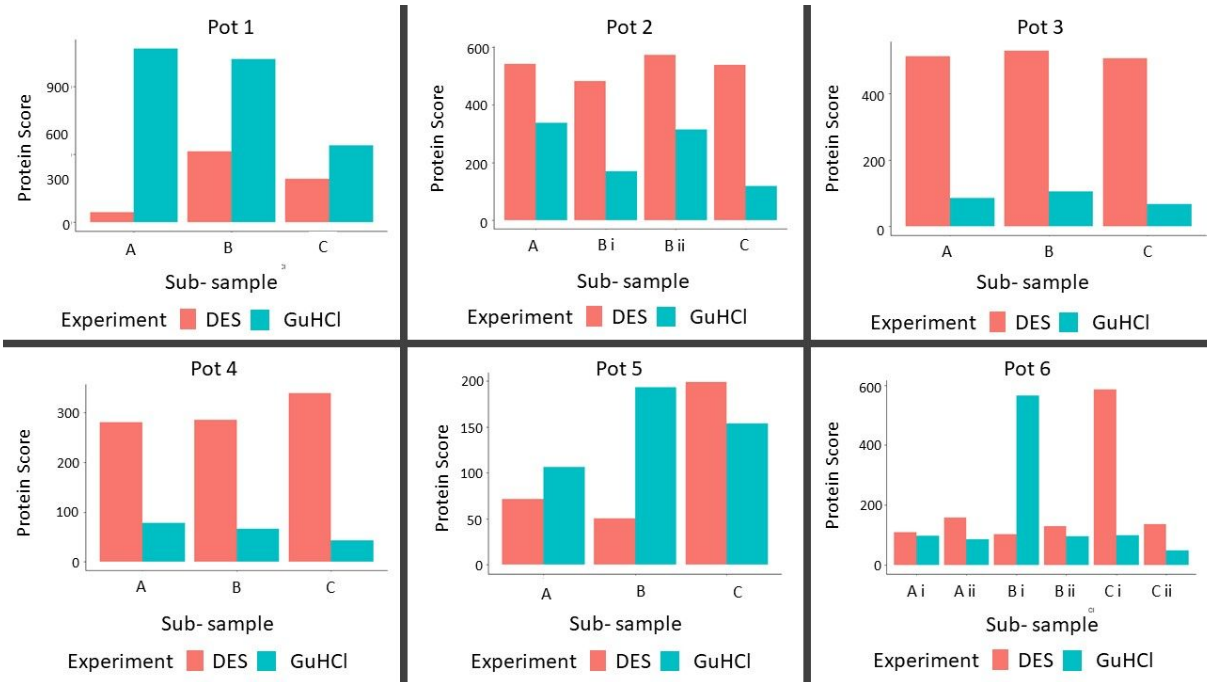

Supplementary Fig. 3: Bar charts comparing the abundances (as calculated using Proteome Discoverer) of the proteins with the highest Mascot score in the six samples (each in triplicates) with SwissProt as the reference database. I and ii indicates that different proteins had the highest score in the GuHCl and the DES extraction. Troponin C (*Anguilla Anguilla*), which was observed in sample 1B in Mascot, was not found using Proteome Discoverer, and hence was not plotted.

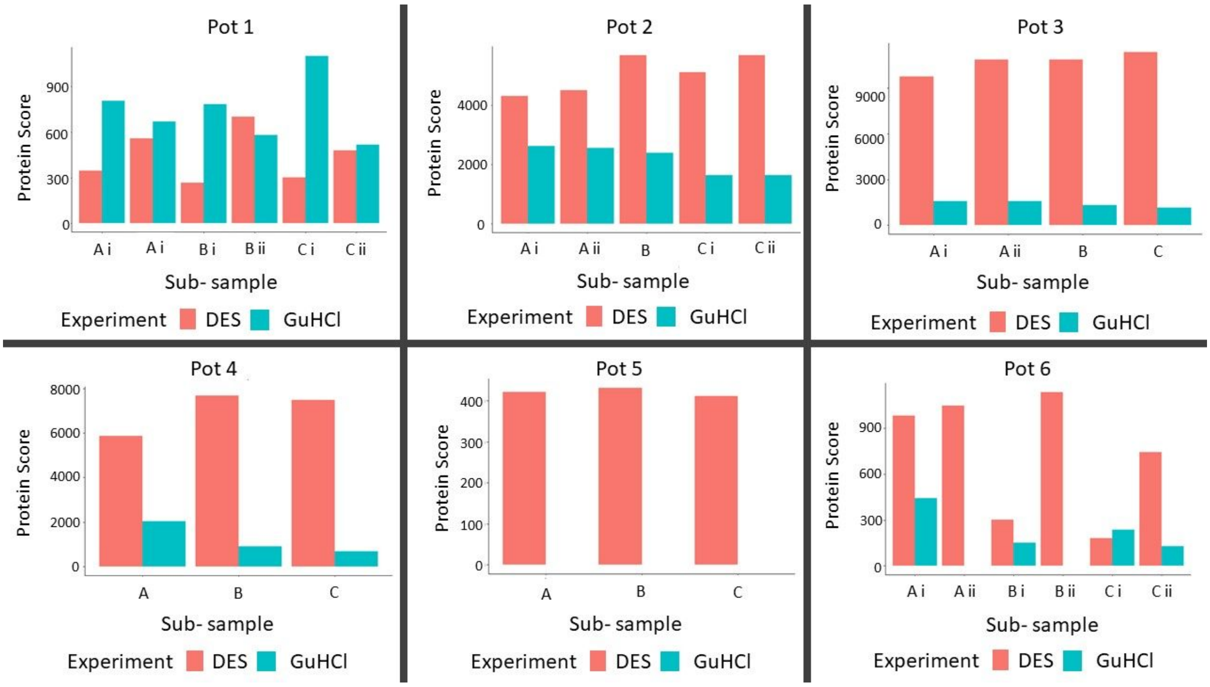

Supplementary Fig. 4: Bar charts comparing the protein scores of the proteins with the highest score in the six samples (each in triplicates) with the custom database as the reference database. i and ii indicates that different proteins had the highest score in the GuHCl and the DES extraction.

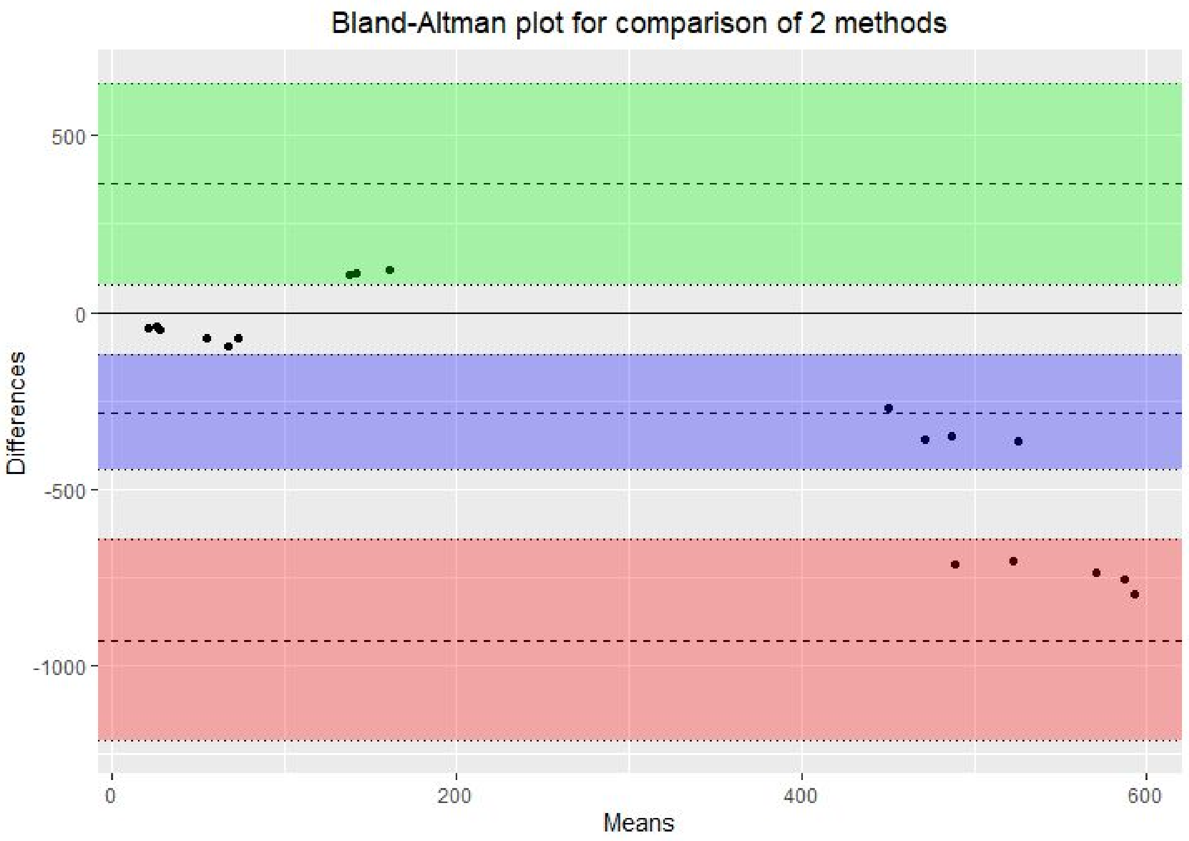

Supplementary Fig. 5: Bland-Altman plot comparing the total number of peptides identified in the samples using the GuHCl and DES-based approach. The mean difference (bias) was -282 where the negative value indicates that the GuHCl-based methods furnished fewer peptides than the DES-based method.

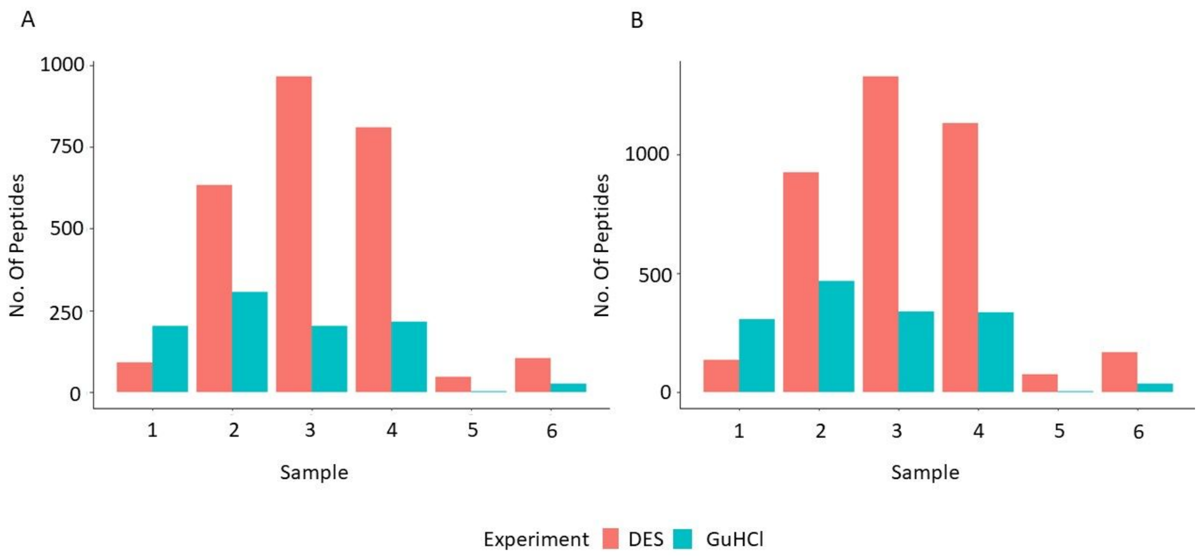

Supplementary Fig. 6: The number of peptides with score above the identity (A) or extensive homology (B) threshold identified in the samples with the custom database as the reference database

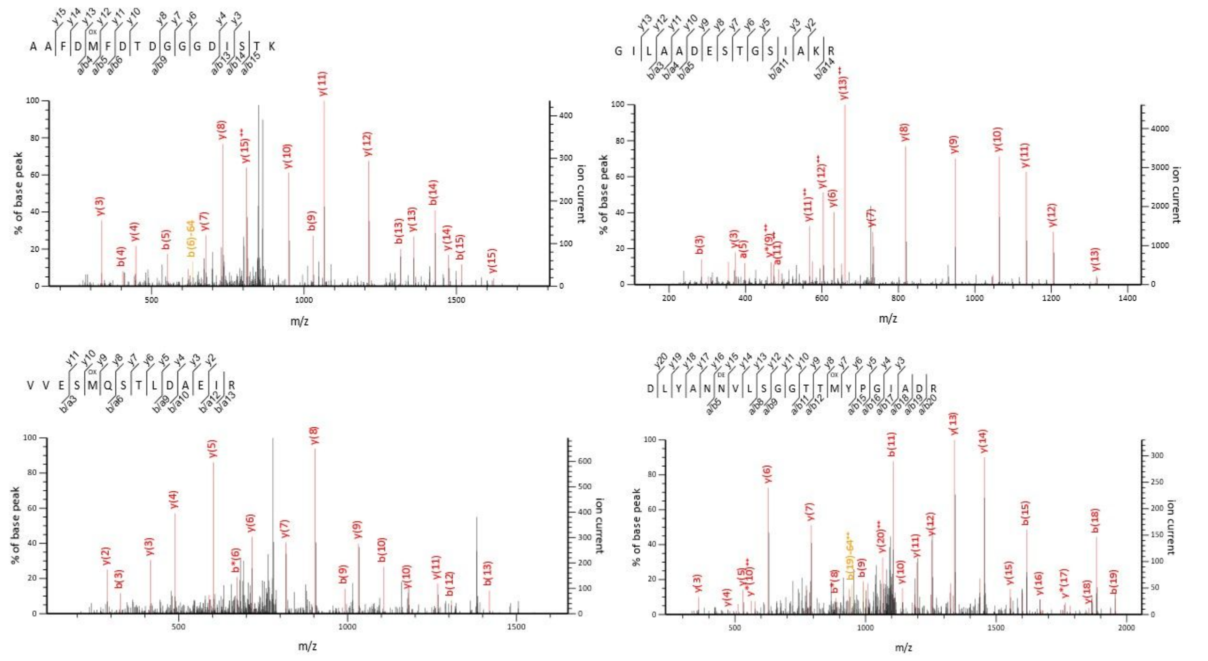

Supplementary Figure 7: Some tandem spectra of peptides identified to be from proteins which were identified in Mascot as belonging to organisms other than the ones processed in the pots. From top left in clockwise order, the peptides are from Troponin C (*Anguilla anguilla*, 1C) Fructose-bisphosphate aldolase A-like (*Sinocyclocheilus anshuiensis*, 2B), Actin alpha skeletal muscle (*Sinocyclocheilus anshuienseis*, 4B) and Myosin 1B-like (*Aythya fuligula*, 4A) respectively.

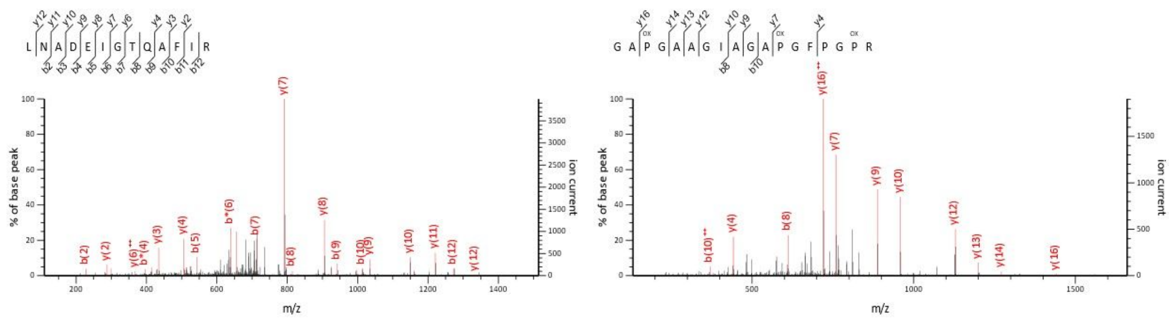

Supplementary Figure 8: Some tandem spectra of peptides identified to be from proteins which were identified in Mascot and belonged to organisms processed in the pots. The peptides shown are from Hemoglobin beta (*Esox Lucius*, 1C) and Collagen alpha 1(I) (*Esox Lucius*, 1A).

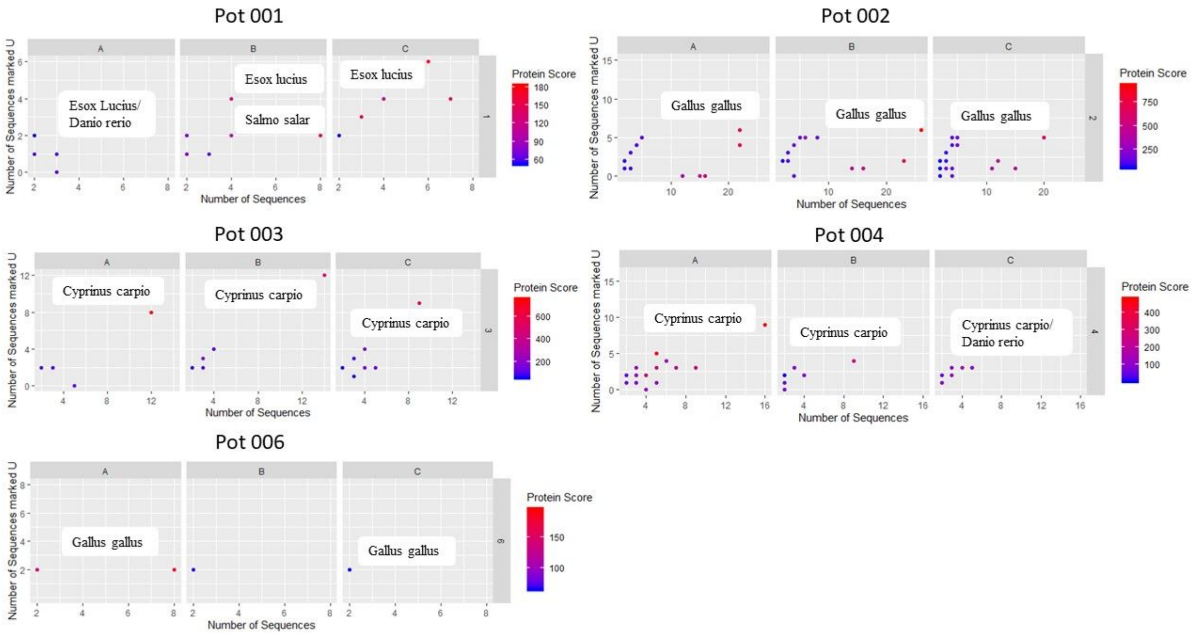

Supplementary Fig. 9 : Scatter plots showing the number of sequences marked U against the total number of sequences. The species of the protein with the highest number of sequences marked U have been mentioned. (SwissProt)
